# Video Gaming and Psychological Well-Being Among Sexual and Gender Diverse Youth in Canada, the United States, Mexico, the United Kingdom, and Australia: Protocol for a LEVEL UP! Cross-Sectional Survey and Screenshot Elicitation Interview Study

**DOI:** 10.2196/95804

**Published:** 2026-09-24

**Authors:** Dane Marco Di Cesare, Ashley Brooks, Kaitrin Doll, Lauren McInroy, Ignacio Lozano-Verduzco, Juan Carlos Mendoza-Pérez, Daragh T McDermott, Yael Perry, Jason Schaub, Paul Byron, Edward J Alessi, Shelley Craig

**Affiliations:** 1 Department of Educational Studies, Brock University, 1812 Sir Isaac Brock Way, St. Catharines, ON, L2S 3A1, Canada, 1 9056885550 ext 5777; 2 Factor-Inwentash Faculty of Social Work, University of Toronto, Toronto, ON, Canada; 3 School of Social Work, Ohio State University, Columbus, OH, United States; 4 Ajusco Unit, National Pedagogic University, Mexico City, Mexico; 5 Department of Public Health/Faculty of Medicine, National Autonomous University of Mexico, Mexico City, Mexico; 6 School of Social Sciences, NTU Psychology, Nottingham Trent University, Nottingham, Nottinghamshire, United Kingdom; 7 The Kids Research Institute Australia, University of Western Australia, Perth, Western Australia, Australia; 8 School for Policy Studies, University of Bristol, Bristol, Avon, United Kingdom; 9 School of Communication, University of Technology Sydney, Sydney, New South Wales, Australia; 10 School of Social Work, Rutgers, The State University of New Jersey, New Brunswick, NJ, United States

**Keywords:** video games, mental health, well-being, lesbian, gay, bisexual, transgender/transsexual, queer, and other minority sexual orientations and gender identities, LGBTQ+

## Abstract

**Background:**

Sexual and gender diverse (SGD) youth experience unique minority stressors that can have a significant impact on their well-being. Many SGD youth use digital technologies to offset these stressors and develop resilience. To date, the role of video gaming in the well-being of SGD youth has not been explored.

**Objective:**

The objectives of the LEVEL UP! study are to (1) investigate the motivations, preferences, behaviors, and experiences of SGD youth as they relate to video game playing and engagement in online video gaming communities; (2) explore how SGD people are represented in video games and how these portrayals are appraised by SGD youth video gamers; (3) identify how video games can support protective factors and processes that contribute to resilience among SGD youth video gamers; (4) develop recommendations and resources (eg, affirming media guides) to make video gaming safer for SGD youth; and (5) demonstrate the utility of video game screenshots as a novel and ecologically valid data source for eliciting discussion from SGD youth about the significance of video gaming in their lives.

**Methods:**

A multipronged, targeted, web-based recruitment approach (social media–based convenience sampling, collaborating with video gaming influencers, Prolific panel recruitment) will be used to recruit approximately 2500 SGD youth aged 14 to 29 years in Canada, the United States, Mexico, the United Kingdom, and Australia to complete a mixed methods, cross-sectional survey hosted on Qualtrics. Key quantitative variables of interest include video gaming habits, preferences, play styles, and the psychological impacts of positive and negative video gaming experiences. Qualitative survey data sources include a brief screenshot elicitation prompt, recall of video gaming experiences, and appraisal of video game content. Canadian participants sampled from the survey will be invited to participate in a follow-up digital screenshot elicitation interview over Zoom to further elaborate on these experiences. Proposed quantitative analyses include exploratory factor analysis, one-way ANOVAs, correlational analyses, and multivariate regressions. Anticipated qualitative analyses include multimodal coding of survey screenshot elicitation data and constructivist grounded theory analysis of the digital screenshot elicitation interview data. Explanatory and convergent mixed methods analyses combining quantitative and qualitative findings are also planned.

**Results:**

The LEVEL UP! study was funded in March 2022, and data collection is expected to occur between October and December 2026, with initial results published in 2027.

**Conclusions:**

LEVEL UP! will provide crucial insight into an underresearched digital subculture that may support the resilience of SGD youth. Opportunities for knowledge translation targeting SGD youth, caregivers, and video game industry professionals are suggested.

## Introduction

### Sexual and Gender Diverse Youth and Minority Stress

Sexual and gender diverse (SGD) youth who identify as lesbian, gay, bisexual, transgender, queer, or other gender or sexual or romantic orientations experience unique minority stressors that can have major impacts on their well-being on top of everyday stressors [1]. Stressful experiences—such as rejection by family due to their SGD identity [2,3]; adverse childhood events, including emotional abuse and neglect related to identifying as SGD [4]; homophobic or transphobic bullying or harassment at school [5,6]; and discrimination by health care providers [7]—can contribute to an overall hostile social environment for SGD youth, which may increase the incidence of mental illness, substance abuse, self-harm, and suicide in this cohort [8]. Furthermore, youth spans multiple developmental milestones, including adolescence, emerging adulthood, and young adulthood [9], which are critical periods during which such mental and behavioral health concerns commonly have their onset.

Despite these heightened risks, SGD youth also evidence tremendous fortitude in the face of adversity. These individual differences can be attributed, in part, to resilience (the ability to adapt constructively to risk exposure, including threats to well-being) [10,11]. Resilience among SGD youth can involve promotive and protective factors and processes, including accessing support networks (eg, supportive family), services (eg, counseling), and using personal traits and characteristics (eg, help-seeking [12]). However, minority stressors at the individual and structural levels (eg, being disowned by family, poor access to competent health care providers) may render certain formal supports infeasible, undermining the ability of SGD youth to leverage factors and processes that can support their coping. As such, SGD youth may develop and practice resilience in unique ways to adapt to these negative circumstances.

### Digital Technologies and Psychological Well-Being

Increasingly, video games have been recognized as a resource for building resilience among youth generally. Gameplay supports stress and emotion regulation, fosters a sense of achievement and agency, and offers avenues for social connection through cooperative and multiplayer formats, with many young people reporting that they use games specifically to manage stress and negative affect [13]. Evidence suggests that digital technologies (eg, social media and apps) can provide a crucial source of informal personal support for SGD youth—and especially for those experiencing significant risk in their offline environments [14-16]. SGD youth have been shown to use digital technologies to support emotion regulation [16], find and build SGD-affirming online communities [17], reduce isolation during the COVID-19 pandemic [18-20], access formative information relating to SGD identities [16,21], and engage in political mobilization [22]. Together, these digital processes and actions generate positive emotional growth, which constitutes a form of “digital resilience” [12].

Most of the research in this area to date has focused on studying website use among SGD youth, including social media sites, apps, streaming services, and multimedia such as TV and film, to support their resilience. Yet evidence points to the popularity of video gaming among youth in the United States, Canada, Mexico, Australia, and the United Kingdom, which surpasses that of other forms of media consumption, such as listening to music, as a primary leisure activity [23-28]. A recent synthesis of the emerging literature suggests that video games could be a source of resilience for SGD youth, indicating that their use supports identity development, coping skills, social support, and civic engagement; however, these early findings are based primarily on qualitative work [29]. SGD youth report using gameplay to cope with stress and anxiety, to exercise control and safety through character customization and storyline choices, and to build and maintain friendships both online and in person [30]. Given the additional burden of minority stress on top of the everyday stressors that affect all youth, video games may represent a uniquely valuable resource for SGD youth.

Although some qualitative research triangulates qualitative interviews with video game footage or screenshots to generate immersive insights into psychological processes related to video game use, this is currently limited to (1) self-interviewing [31], (2) eliciting reactions and conversation from researcher-selected screenshots or gameplay sessions [32,33], (3) analyzing secondary data such as fan-made gaming videos [34], (4) screenshots from single games [34,35], and screenshots focused on a single subject (eg, avatars) [35]. There is presently no methodological guidance explaining how video game screenshots can be integrated into digital photo elicitation interviewing to empower participants to cocreate the interview experience [36,37], and how video game screenshots can be systematically analyzed in multimodal qualitative research [38]. Furthermore, alongside the potential for digital resilience, there also exist risks such as bullying and harassment by other players [39] and problematic and addictive video gaming behavior [40] that may temper these benefits, necessitating further qualitative and quantitative investigation.

### Objectives

This international study, LEVEL UP!, will be conducted in Canada, the United States, Mexico, the United Kingdom, and Australia to (1) investigate the motivations, preferences, behaviors, and experiences of SGD youth as they relate to video game playing and engagement in online video gaming communities; (2) explore how SGD people are represented in video games and how these representations are appraised by SGD youth who play video games; (3) identify how video games may support promotive and protective factors and processes that develop resilience among SGD youth who play video games; (4) develop recommendations and resources (eg, affirming media guides) to promote healthier and safer video gaming for SGD youth; and (5) advance the utility of video game screenshots as a novel and ecologically valid data source by developing a systematic screenshot elicitation interview protocol. This paper will outline LEVEL UP!’s research protocol, including study design, recruitment methods, quantitative and qualitative data collection procedures, intended mixed methods analyses, and expected knowledge mobilization outputs.

## Methods

### Study Design and Team

The LEVEL UP! study will adopt an explanatory mixed methods design (QUANT+QUAL) comprising (1) a web-based cross-sectional survey of SGD youth aged 14 to 29 years in Canada, the United States, Mexico, the United Kingdom, and Australia who play video games and (2) a follow-up qualitative semistructured video game screenshot elicitation interview study with Canadian SGD participants aged 14 to 29 years recruited through the cross-sectional survey. A web-based cross-sectional survey hosted on the secure survey platform Qualtrics was deemed an effective method for collecting data from SGD youth who play video games because both groups (ie, SGD youth and people who play video games) use digital technologies intensively [41].

The LEVEL UP! study team is composed of (1) 2 coprincipal investigators, (2) a research coordinator, (3) a doctoral student, (4) a graphic designer, (5) an English-Spanish translator, and (6) 2 consulting faculty members from institutions in Mexico. Additionally, the study team—based primarily in Canada—will consult with and provide study updates to partnered faculty members at institutions in the United States, Mexico, the United Kingdom, and Australia for support with research tasks such as study design (eg, wording of demographic questions to capture local conventions), recruitment efforts, data analysis, and manuscript preparation. The study team will further be supported by 7 research assistants—supervised by the research coordinator—from various degree levels (ie, bachelor’s, master’s, and doctoral). These research assistants will complete research tasks including preparing literature reviews, constructing the online survey, study recruitment, conducting qualitative interviews, mixed methods data analysis, and drafting dissemination materials. The study team includes SGD hobbyist video game players (ie, one of the coprincipal investigators, the research coordinator, and some research assistants), as well as team members with expertise in research involving SGD youth. SGD youth will be further involved in the study through a preexisting international youth advisory board [42] of up to 12 SGD youth, who will inform study design, measurement selection, recruitment strategies, interview schedule design, data analysis, and knowledge dissemination. The international youth advisory board was created by the larger INQYR Partnership in 2002.

This research was funded by the Social Sciences and Humanities Research Council of Canada through an interdisciplinary and multilingual international research partnership [43]. The partnership examines the resilience of LGBTQIA2S+ (lesbian, gay, bisexual, transgender, queer, intersex, asexual, Two-Spirit plus) youth through technology-engaged research, aiming to deepen understanding of youth resilience across multiple regional contexts in an increasingly digitized world. This is pursued through methods such as digital photo elicitation and other technology-focused research with LGBTQ+ (lesbian, gay, bisexual, transgender/transsexual, queer, and other minority sexual orientations and gender identities) youth. The goals of the grant informed the design of this research.

### Participants

The study eligibility criteria are as follows: (1) aged 14 to 29 years; (2) self-identifies as SGD; (3) currently living in Canada, the United States, Mexico, the United Kingdom, or Australia; (4) plays video games regularly; and (5) able to participate in English or Spanish. A multipronged purposive recruitment approach will be used to recruit participants who meet these criteria.

Digital posters and recruitment emails will be sent a minimum of 3 times to a list of over 1000 national and regional agencies providing direct services to SGD youth (eg, La Paz Es Diversa), advocacy organizations (eg, It Gets Better), clubs (eg, Vancouver Gaymers), news outlets (eg, Gayming Magazine), and research organizations (eg, The Kids Research Institute), all related to SGD and video gaming populations and based in Canada, the United States, Mexico, the United Kingdom, and Australia. This will be supplemented with direct messages to moderators of Facebook groups (Meta Platforms, Inc), Subreddits (Reddit, Inc), and Discord servers (Discord Inc) devoted to SGD or to video gaming topics, requesting permission to post recruitment materials in their communities. Social media recruitment posters will also be regularly shared on the laboratory’s social media pages (X [formerly Twitter; X Corp], Facebook, TikTok [ByteDance Ltd], Instagram [Meta Platforms, Inc], LinkedIn), and aspects of the poster will be changed over time to increase engagement with populations for which we may need to seek more input (ie, including images of racialized and transgender video game characters). On social media, the potential for posts to be tagged and reposted by additional followers may result in increased reach. Further, post reach (ie, the number of unique people who see the advertisement) will also be boosted with paid social media advertisements, which have the potential to produce a large collective social media reach estimated to be over 140,000 people. Additionally, the study team will leverage personal and professional networks to share the study among academics and video game designers.

To enhance the credibility and trustworthiness of the study, the LEVEL UP! institutional and funder logos will be integrated into the recruitment materials [44]. The study team also aims to recruit 5 or more “LEVEL UP! Champions,” SGD video game and variety streamers who are active on social media platforms, including the online livestreaming service Twitch (Amazon). Prospective champions will act as volunteer social media influencers to promote the study to their audiences by reposting recruitment posts and advertising it during selected livestreams. In return, champions will be offered (1) a space on the laboratory’s website to promote their social media channels and provide a quote speaking to their commitment to creating safer online spaces for SGD people; (2) social media posts highlighting their support of the study and promoting their streams; and (3) spotlights in an SGD-affirming video game media guide emanating from the study findings. The use of social media influencers for online health research recruitment has been noted to support recruitment from hard-to-reach communities [45].

Finally, recruitment for the survey will be incentivized with the option to enter an email address into a prize draw to win either (1) one CAD $250 (approximately US $182) Amazon gift card, (2) one of 2 CAD $100 (approximately US $73) Amazon gift cards, or (3) one of 10 CAD $50 (approximately US $36.5) Amazon gift cards. Equivalent value gift cards will be offered across the 5 countries. The survey will be further incentivized through nominal direct payments to participants recruited through an online prescreened participant panel, *Prolific*, a bespoke alternative to “crowdwork” platforms such as Amazon Mechanical Turk for online research recruitment [46]. Given Prolific participants’ regular participation in online surveys and the growing potential of participant fraud through such platforms, no more than 15% of the participants will be recruited from this source, and the data source will be recorded for sensitivity analyses. Eligible participants will additionally be offered a CAD $50 (approximately US $36.5) Amazon gift card to participate in an interview after completing the survey.

### Data Collection: Survey Measures

Participants will complete a 30- to 45-minute web-based survey in Qualtrics [47]. The survey (Multimedia Appendix 1), available in English and Spanish, contains 6 sections of questions following the consent form and a CAPTCHA. To aid participant understanding and reduce fatigue, each section of the survey will be clearly described and features visually unique headers to differentiate them.

Section 1 of the survey is designed to collect essential participant demographics, including age, country of residence, and country of birth (if not the same); gender identity (all that apply); sexual orientation (all that apply); level of outness as an SGD person (nobody, everybody, or some, with follow-up questions about the people they are out to if “some” is selected); racial and ethnic identities (all that apply, with free text boxes to further define); and disability and neurodiversity status (all that apply, diagnosed or otherwise) using 11 harmonized categories across all study regions to characterize the nature of the disability (eg, vision impairment, mental health condition, and mobility issues). Drawing from previous survey design, participants who select multiple gender identities or sexual orientations will be asked to select the one that is most important to them for use in quantitative analyses [48]. In addition, following regionally specific consultation, certain gender identity response options (ie, Two-Spirit, Muxe, Brotherboy, and Sistergirl) will only be shown to participants from countries where those identities are culturally relevant using display logic. Participants will also indicate where they heard about the survey. Using a visual data collection methodology developed by one of the coprincipal investigators [12,36,37], Section 1 contains an optional screenshot elicitation prompt whereby participants will be instructed to “upload a screenshot from a video game that makes you feel strong, validated, and proud,” explain why they chose their selected screenshot, and identify whether they took the screenshot themselves or retrieved it from elsewhere. This innovative data collection approach aims to collect multimodal data about the participants’ video gaming and help them articulate video game scenes or moments that affirm their identity or foster resilience.

Section 2 includes questions constructed by the research team about the age at which participants started playing video games; the actual and ideal number of hours spent playing video games in a typical week; technologies used to play video games; motivations for playing video games, using items adapted from the Gaming Motivation Inventory, simplified for youth audiences [49]; and favorite genres. Participants will be asked whether they played single-player games or multiplayer games, livestreamed themselves playing video games to an audience, and accessed online gaming communities.

Questions in Section 3 ask participants whether they identify with or relate to video game characters or storylines (and, if so, to describe why); their appraisal of the frequency and quality of SGD representation in video game characters, themes, and character customization; and how satisfied they are with characters they can select and create. To identify the psychological, social, and behavioral impacts of negative and positive experiences with video game content, 2 scales used previously with SGD youth inform this survey, which were heavily adapted to account for the gaming experience and context. Specifically, the Social Media Benefits Scale [50] and the Digital Microaggressions Scale [51,52] are used, with questions modified in collaboration with the youth advisory board to target video gaming contexts, asking, “Thinking about all of the negative/positive experiences with video game content, how much were you impacted in the following ways?” Items include, “I felt embarrassed” and “I felt comfortable,” with responses scored on a 5-point Likert scale from 1 (“I was not affected this way at all”) to 5 (“I was very much affected this way”) and total scores calculated.

Only Sections 4 and 5 will appear to participants who report playing multiplayer games or using online video game communities in Section 2. Section 4 asks participants about microaggressions they experienced or witnessed in these settings (eg, “using the wrong personal pronouns or name to reference you/others”); the frequency of those experiences, where they occurred, who the target was, and the psychological, social, and behavioral impacts. Section 5 is structured in the same way but instead asks about allyship they experienced or witnessed (eg, “someone asked what pronouns they should use for me/another person”) and is presented after negative experiences to potentially counteract distress experienced while recalling negative experiences in Section 4. The impacts of microaggressions and allyship experiences will be measured using the same adapted scale items from Section 3.

The final section of the survey, Section 6, contains information about the prize draw and an optional field for participants to enter an email address if they wish to participate. Finally, Canadian participants will be shown information about an optional screenshot elicitation interview (detailed in the following section) and will receive an invitation to participate, sent to their provided email address. The full survey will be translated from English to Spanish and back-translated into English by a translator, who will confer with the Spanish-speaking youth advisory board members to collaboratively agree upon changes [53].

### Digital Screenshot Elicitation Interviews

Digital screenshot elicitation methods constitute a novel digital adaptation of photo elicitation interviewing, an arts-based interviewing technique in which participants are instructed to take photos related to prompts associated with a central research question [36,54,55]. These photos are then integrated into and discussed during a semistructured interview to generate richer narratives and new insights that may not have been gleaned from typical interview methods [54,56]. Because participants take their own photos, their agency in the research process is enhanced, making this approach suitable for participants who experience marginalization and disempowerment [57,58].

Screenshot elicitation interviewing draws on digital photo elicitation methodology and adapts it to video games. Building on the screenshot elicitation prompt used in Section 1 of the survey (to answer a question), the screenshot elicitation interviews will be used to start new inquiries, expanding upon survey data with additional depth, insights, and nuance. For SGD youth, who may be accustomed to having their experiences filtered through adult or heteronormative interpretive frames, selecting their own screenshots shifts interpretive control to the participant, allowing them to define which aspects of their gaming experience (representation, identity exploration, community, etc) are worth discussing rather than responding only to researcher-generated questions. The use of screenshots will also provide visual anchors for discussion during the interviews, making it easier to understand games, characters, features, and contexts participants want to discuss. This will also aid in the analysis, as the team is composed of both gamers and nongamers. It is anticipated that screenshot elicitation will provide insight into how youth interact with video games, similar to research that explores the role of digital technologies in immigration and social mobility [59] and in managing mental health [60,61]. As previously discussed, while some other research has triangulated textual data and video game screenshots [62], LEVEL UP! will be, to our knowledge, the first study to fully integrate video game screenshots into a systematic screenshot elicitation methodology.

The digital screenshot elicitation interviews will be limited to Canadian participants only, as much of this research is focused in the United States and the primary research team is in Canada. Canadian participants who express an interest in participating in an interview will be screened using the demographic data supplied in Section 1 of the survey to ensure the sample represents a diverse range of ages, gender and sexual identities, and racial and ethnic identities. Thus, we aim to recruit between 35 and 50 participants to capture a variety of perspectives from participants with differing intersectional identities. Furthermore, as participants complete interviews, the research team will use participants’ identities to prioritize any identities currently not represented when contacting additional participants. Invited participants will be contacted by 1 of 2 members of the study team and instructed to select between 5 and 10 screenshots from video games or video game communities related to the following three prompts: (1) your gaming preferences; (2) your experiences with SGD characters, storylines, or anything related to SGD representation in video gaming; and (3) your experiences with or in video gaming communities as a member of the SGD community. Once the screenshots are received, the interview will be scheduled with either the principal investigator or one of the coinvestigators based on availability. Prior to the interview, screenshots will be labeled with the name of the video game to allow for greater ease and efficiency in screen sharing during the interviews. A protocol document with naming conventions and directions will be made available to the interviewers. Further, any identifying information (ie, gamertags) will be redacted from the screenshots prior to the interview to protect confidentiality when these materials are coded.

Using a semistructured interview style and the methods and protocol developed in previous digital photo elicitation studies [37,38], interviews will be conducted online using the teleconferencing software Zoom [63], which has been used effectively to conduct online photo elicitation interviews elsewhere [36,37]. The coprincipal investigators chose Zoom, in part, due to the ease with which the interviewer could display screenshots for both parties to view and discuss simultaneously. Other benefits of using Zoom included live captioning, privacy settings that hide participants’ names in the recording, and the ability to record and save interview audio and video files.

At the start of the interview, participants will be taken through the consent process and told that they are free to skip any questions they do not wish to answer or to stop the interview at any time. Interviews will only begin after participants have provided consent.

The interview protocol is carefully designed with questions organized into 5 themes: gaming preferences (including favorite games and genres), participation in gaming communities, in-game SGD representation, and impact of negative experiences and positive gaming experiences. Within each theme, related questions will be asked, and the 5 to 10 screenshots sent in advance will be shared and discussed. This will be free-flowing and varied based on the individual participant’s experiences. Participants will be told that if any of their screenshots pertain to the theme or the question they are asked, they can ask the interviewer to share the corresponding screenshot when discussing a particular question. Additionally, if the interviewer feels a particular screenshot fits into the context of a theme or a participant’s response, they can ask the participant for permission to share that screenshot for discussion at that time. If any screenshots remain at the end of the interview, the interviewer will pull them up, one at a time, to discuss. Before moving to a new theme, participants will be asked to identify any additional relevant screenshots. Question prompts regarding the screenshots relate to context, reasons for selection, emotions connected to the screenshot, and anything else the participant deems important or worth sharing. With questions specific to each theme, interviewers will move between the semistructured questions and screenshots according to the participant’s comfort level, the questions’ applicability, and the conversation flow. They will also have the freedom to ask follow-up questions as appropriate. Using the interview protocol will aid interviewers in taking a consistent approach, thus minimizing inconsistencies in data collection.

### Ethical Considerations

Prior to completing the online survey, all participants will have the option to read a text-based consent form; watch a short, animated video created using Vyond [64], which explains each consent statement in a more engaging multimedia format [36,37]; or view both. These materials were checked for grade-school-level readability using Readable [65] to ensure they were comprehensible to the youngest participants. Both informed consent processes will include standard elements, including information about what the study is about, what participation would involve, risks and benefits of participation, approximately how long the study will take, participants’ right to withdraw at any time, privacy and confidentiality information, links to regionally specific support resources, and the principal investigator’s contact information. Participants who provide their own screenshots in the survey will also need to provide consent to share anonymized screenshots (ie, in written publications, presentations, on the laboratory’s website, on the laboratory’s social media platforms; all of these, or none of these).

In accordance with the approval from the research ethics board, parental consent will not be required for participants aged 14 to 17 years to remove the requirement for closeted SGD youth participants to disclose their SGD identities to participate [66] and to reduce the risks of participation for SGD youth who may have unsupportive parents or guardians [67]. Participants will not be required to provide personally identifiable information to complete the survey. Respondents who wish to enter the postsurvey prize draw or indicate interest in a follow-up interview may voluntarily provide an email address through a separate form not linked to their survey responses. This form will also include instructions for creating a throwaway email address for the purposes of the prize draw, using Temp-Mail (Privatix Ltd), to ensure privacy. Any email addresses collected for the prize draw or interview recruitment will be stored separately, used only for these stated purposes, and deleted once these procedures are completed. Data will be collected through a secure institutional Qualtrics account, stored on secure, password-protected institutional servers, and accessible only to authorized members of the research team.

Certain sections of the survey focusing on negative video gaming experiences (eg, being the target of a homophobic joke about sexual orientation or gender identity) are preceded by a content advisory, and, upon concluding the survey, a link to regionally specific support resources will be provided. The study protocol received research ethics board approval from the University of Toronto Health Sciences Research Ethics Board (approval number: 41573). This approval explicitly covers international recruitment across all 5 countries (Canada, the United States, Mexico, the United Kingdom, and Australia).

### Sample Size and Power Calculations

The target survey sample is approximately 2500 participants across the 5 participating countries, with approximately 500 participants per country, although equal country sample sizes cannot be guaranteed given the study’s purposive, nonprobability recruitment strategy. For the planned linear multiple regression analysis, power calculations in G*Power (developed by the University of Düsseldorf, Germany) assuming α of .05, power of 0.80, a small effect size *f*^2^ of 0.02, and 3 predictors indicate a minimum sample size of 550 participants. For cross-country comparisons, power calculations in G*Power assuming α of .05, power of 0.80, a small effect size *f*^2^ of 0.1, and 5 groups suggest a minimum sample size of 1200 participants. The larger recruitment target of 2500 participants was selected to additionally accommodate data loss resulting from incomplete or potentially fraudulent responses (assuming up to 50% could be fraudulent based on prior research [68]) and to facilitate exploratory comparisons across demographic groups (eg, sexual orientation and gender identity). An interview sample size of 40 participants is expected, assuming approximately 5% to 10% of Canadian survey participants also participate in an interview.

### Proposed Quantitative Analyses

Quantitative data will be analyzed using SPSS version 27. First, to minimize the impact of fraudulent responses and survey bots on data integrity, a priority-based coding process will be undertaken based on recent data-cleaning recommendations to identify and remove such responses [69-71]. Responses will be coded by 2 trained coders as potentially fraudulent if they (1) are started within 1 minute of another response; (2) contain nonsense or irrelevant qualitative answers; (3) report a country of residence that does not correspond with their IP address; (4) contain duplicative qualitative answers, IP addresses, or email addresses; (5) have a completion time below the 10th percentile of the median response time; (6) report factually inconsistent information at different points in the survey (eg, mismatched age); and (7) provide illogical information (eg, an implausible amount of gaming per week). The coding data will be analyzed using hierarchical and k-means cluster analyses to group responses with similar potential fraud characteristics into distinct clusters [68], followed by a human review of borderline cases by the 2 coders. Data missingness will be explored using Little’s Missing Completely at Random test, with multiple imputations used to replace missing data as appropriate.

Quantitative analyses will aim to answer the following research questions (RQs): RQ1: What are the video gaming habits, motivations, preferences, and experiences of SGD youth? RQ2: What underlying dimensions characterize the positive and negative psychological, social, and behavioral impacts of video gaming among SGD youth? RQ3: How do the microaggressions and bystander allyship in multiplayer gaming settings relate to video gaming characteristics and impacts? and RQ4: How do video gaming characteristics and impacts differ across countries, demographics, and levels of outness?

Frequencies will be calculated to characterize the gaming habits and preferences of SGD youth (eg, single-player vs multiplayer, reasons for playing, genre preferences) (RQ1). Following preliminary checks for data suitability (eg, Kaiser-Meyer-Olkin tests, Bartlett test of sphericity), exploratory factor analysis will be performed on the video gaming benefits and impacts items to create composite measures, and mean scale scores will be computed (RQ2). Invariance analysis across participants who take the survey in English or Spanish will also be conducted to test for measurement equivalence (RQ2). Counts of the occurrences of microaggressions and allyship experiences in multiplayer settings will be calculated and summed to create composite measures, with higher scores indicating more experiences of microaggressions and allyship. Counts of the gaming settings in which each behavior occurred will be calculated, and the frequency of microaggression and allyship experiences will be averaged for each setting to create measures of the average frequency with which these experiences occur across different settings (RQ3). These measures will be correlated with the impacts of those experiences, and impacts and experience frequency will be compared across multiplayer settings to understand how multiplayer video gaming relates to risk and resilience among SGD youth (RQ3). Further regression analyses will examine the level of outness, multiplayer setting, and country of residence as predictors of experienced and witnessed microaggressions and allyship, with weekly time spent playing video games as a covariate (RQ3). One-way ANOVAs will be used to compare the video gaming benefits and impacts scale between different countries to explore how sociocultural context may influence these processes (RQ4). Further subgroup analyses (eg, across sexual orientation and gender identity categories) will be conducted, with multiple-testing handled using Bonferroni or Tukey Honestly Significant Difference–corrected significance values (RQ4). Sensitivity analyses will be performed to detect or rule out potential differences between the convenience and Prolific subsamples and among different convenience sampling sources identified in Section 1 of the survey.

### Proposed Qualitative Analyses

Interview video recordings and transcripts, screenshots, and open-ended survey question responses will be analyzed using a constructivist grounded theory approach [72] to inductively generate a theory of how SGD youth use video games to support their well-being and to answer RQ5: How do SGD youth use video games to support their psychological well-being? Prior to analysis, coders will receive training on constructivist grounded theory and multimodal coding in NVivo 15 (Lumivero), a qualitative data management software chosen for its ability to integrate a variety of data modalities relevant to the study (eg, text, images, audio, video). The interview data will be analyzed using a 4-step multimodal data analysis protocol [36,38]: (1) initiating understanding by reading transcripts, inspecting screenshots, and watching interviews; (2) open coding to determine an initial set of codes; (3) axial and focused coding to refine and abstract the codes into larger analytical concepts; and (4) finalizing the codes and generating themes in 10 monthly 1-hour meetings with the coding team. To facilitate a systematic and integrative analysis, the 3 data modalities will be analyzed by their respective coders in a nested sequential fashion (ie, screenshots, then transcripts, then screenshots again, then the interview recordings) to gradually add layers of complexity to the data [36,38]. First, screenshots will be coded for demographic and visual details, including characters, character interactions, scenery, text, and gameplay, to capture their content. During line-by-line coding of the transcripts, coders may also add more abstract codes (eg, relating to the game’s themes) to the screenshots as participants discuss and interpret them in the transcript. Likewise, the interview recordings add complexity to the transcripts (eg, facial expressions, vocal tone, video game paraphernalia) that provide analytic insights that neither the screenshots nor transcripts confer. As such, this analytic process enhances the constant comparative method in constructivist grounded theory because it encourages comparisons between different data modalities as well as between different codes and between or within participants [36,38]. This process will be further enhanced by the use of “see-also links” in NVivo 15 to link sections of interview transcripts and recordings to screenshots as the participants talk about them. Screenshots and qualitative answers submitted in the survey’s screenshot elicitation question will also be analyzed using a similar 4-step multimodal data analysis protocol. This analytic process will be systematized to answer RQ6: How can video game screenshots be integrated into digital photo elicitation interviews and multimodal qualitative analysis?

### Proposed Mixed Methods Analyses

Consistent with an explanatory mixed methods approach [73], new research questions based on the quantitative findings will be generated that use qualitative data to explain patterns, add depth, and inform subsequent studies. An exploratory mixed methods approach will also be taken by generating counts of the games depicted in video game screenshots and analyzing qualitative patterns across different genres, with results saved in NVivo 15 as file classifications and nodes. A convergent mixed methods approach will also be taken by matching quantitative data (eg, Video Gaming Impacts Scale) to screenshot elicitation interview data to examine how participants’ narrative accounts and screenshots contextualize, expand upon, and potentially contrast with their questionnaire data using a joint display [74]. Integrating quantitative and qualitative findings will provide a richer understanding of participants’ well-being related to their video-gaming habits and experiences than either approach alone.

## Results

The LEVEL UP! study was funded in March 2022, and data collection is expected to occur between October 2026 and March 2027, with initial results published in 2027.

## Discussion

### Principal Results

LEVEL UP! will provide novel insights into the digital subcultures of video gaming and how these may influence the psychological well-being of SGD youth. It is expected that findings will show that video gaming influences SGD youth well-being both positively (eg, role-playing SGD aspects of self) and negatively (eg, distress caused by harassment in multiplayer and community gaming contexts) and will be mediated by game design (eg, inclusive avatar creation options) and cultural factors (eg, cross-cultural differences in gaming access and meaning). With a target of over 2500 SGD youth participants across 5 countries, this has the potential to be the largest and most diverse study of its kind to date, providing valuable quantitative, qualitative, and mixed methods findings and greatly enhancing the largely qualitative evidence base developed so far [29]. When data collection is complete, the results of the proposed analyses will be shared through various forms of knowledge mobilization, disseminated using channels similar to those used for participant recruitment. For example, counts of video game characters depicted in screenshots and noted by participants will be consolidated into an affirming video game character and media guide containing video game and character spotlights, illustrative quotes, and game recommendations.

### Comparison With Prior Work

The findings of the LEVEL UP! study will build on previous social media research, which has elucidated several technology-mediated ways that SGD youth escape from victimization [75], find identity-affirming information [41], explore SGD identities and self-expression [16,60], develop mutually supportive social relationships [15], and regulate emotions [12]. LEVEL UP!’s expansion of this inquiry into video gaming will be a significant advancement from previous research, as video games have unique design features (eg, storytelling or narrative) that may relate to SGD youths’ psychological well-being differently from that of social media. This distinction is particularly important given proposed and enacted bans on social media and video game use among youth internationally [76]. Furthermore, video gaming among SGD players has largely been studied within humanities disciplines using qualitative methods with participants in North America [29], whereas LEVEL UP! will have wider international and cross-disciplinary relevance across psychology, social work, and media studies through its quantitative, qualitative, and mixed methods design and international and interdisciplinary team.

### Strengths and Limitations

The study design confers several strengths. Like social media screenshots, video game screenshots may be an ecologically valid data source to understand the digital environments in which SGD youth are socialized [77], and eliciting such screenshots may also facilitate the participation of a more diverse sample. For example, neurodiverse SGD youth and those with disabilities may choose to share video game avatars to represent those aspects of their intersectional identities in digital spaces, adding richness to the analysis [78]. Screenshots also require less effort to acquire than photographs, broadening participation to SGD youth who may have less time to participate in research. Furthermore, the international study will elucidate differences and similarities between the participating countries, facilitating both generalizable and local recommendations for public policies or interventions involving video games to positively impact the quality of life of SGD youth.

This project has several limitations. First, the study will be completely online and focuses on a pastime that typically requires technological and financial resources, presenting a participation barrier for SGD youth who lack access to gaming technologies or the internet (eg, those who are low-income individuals, unhoused youth, or those living in remote areas without internet access). The predominantly social media–based sampling strategy may also generate selection bias. As well, the survey is extensive, and the screenshot elicitation interviews will require time for participants to prepare for and participate in, which could be a barrier for SGD youth who do not have time to participate. As with much research involving SGD youth, participants’ responses may be shaped by social desirability or disclosure comfort, particularly given the sensitivity of discussing identity and marginalization with unfamiliar researchers. This may lead some participants to underreport experiences of discrimination or present a more positive account of their gaming experiences than they privately hold. Furthermore, since participation in the screenshot elicitation interview is limited to Canada due to the location of the primary research team, the sample will not be representative of all SGD video game players. Several of the team members play video games regularly, and half of the team does not play video games. This may result in potential intellectual conflicts of interest, as gamers on the team may bring assumptions shaped by their own gaming preferences or genre familiarity, while nongamers may lack the tacit knowledge needed to interpret certain gameplay references or subcultural norms raised by participants. To mitigate this, analysis will be conducted collaboratively across both gamer and nongamer team members, allowing gaming-related assumptions to be checked against outsider perspectives and unfamiliar references to be clarified by those with direct gaming experience. There is also a potential for curation bias as participants self-select which screenshots to bring to the interview, and the resulting data are inherently curated. Participants may avoid images they perceive as too revealing or select ones that present a particular version of their gaming experience, which may limit the depth or authenticity of the insights gathered. Finally, the use of video game screenshots as a data source is a new approach, and one limitation of this approach is that they may contain less “original” information than screenshots from other sources. For example, a screenshot from a mental health app may depict internal processes such as mood and sleep documented by a participant [61], whereas video game screenshots may be more derivative if they depict scenes where no original video game content, such as the player avatar, is present.

### Conclusions

LEVEL UP! is poised to be the largest study of the links between video gaming and psychological well-being among SGD youth to date and the only international study of its kind. The findings generated by this research will provide crucial information about the positive and negative impacts of video games, video gaming communities, and SGD representation in video gaming on the psychological well-being of SGD youth, which will be of value to SGD youth themselves, as well as their caregivers and video game industry professionals aiming to create safer online spaces for SGD youth to connect, have fun, and express themselves. In addition to dissemination through traditional academic channels such as journal articles and conference presentations, there are significant opportunities to translate the findings into various multimedia formats (eg, affirming media guides, digital galleries, and infographics) to equip knowledge users with actionable recommendations for affirming media consumption and for innovating more SGD-inclusive features in video games.

## Supplementary material

10.2196/95804 Multimedia Appendix 1 LEVEL UP! survey.

## Abbreviations

LGBTQ+: lesbian, gay, bisexual, transgender/transsexual, queer, and other minority sexual orientations and gender identities
LGBTQIA2S+: lesbian, gay, bisexual, transgender, queer, intersex, asexual, Two-Spirit plus
RQs: research questions
SGD: sexual and gender diverse
