# Supplementary material for: Video Gaming and Psychological Well-Being Among Sexual and Gender Diverse Youth in Canada, the United States, Mexico, the United Kingdom, and Australia: Protocol for a LEVEL UP! Cross-Sectional Survey and Screenshot Elicitation Interview Study
Source: JMIR Res Protoc. 2026 Sep 24;15:e95804. doi: 10.2196/95804 (PMC13606322; doi:10.2196/95804)
Supplement: Supplementary file 1 [file resprot-v15-e95804-s001.pdf]

LEVEL UP!
An LGBTQ+ Gaming Study

¡SUBE DE NIVEL!
An LGBTQ+ Gaming Study

**LEVEL UP!**<br>**An LGBTQ+ Gaming Study**	**SUBE DE NIVEL!**<br>**An LGBTQ+ Gaming Study**
Please note, due to the complex skip logic in Qualtrics, the question numbers may not be in chronological order or there may be duplicate numbers.	Tenga en cuenta que, debido a la compleja lógica de salto en Qualtrics, los números de las preguntas pueden no estar en orden cronológico o puede haber números duplicados.

Note: An open-ended response to "How did you learn about this study?" will begin the survey.

Nota: La encuesta comenzará con una respuesta abierta a la pregunta "¿Cómo se enteró de este estudio?"

	SECTION 1: DEMOGRAPHICS	SECCIÓN 1: PREGUNTAS DEMOGRÁFICAS
	Thank you! By participating in the LEVEL UP! Project, you are helping us to make gaming a more open place for LGBTQ+ youth!<br><br>**This survey will take approximately 20 minutes to complete.**<br><br>For participating in this research, we would like to thank you by offering you an opportunity to participate in a raffle to win one of 10 $50 CAD (40 USD; 30 GBP; 55 AUD; 819 MXN) Amazon gift cards. More information will be available after you submit the survey.<br><br>In the survey, you will have an opportunity to upload a screenshot from a video game that makes you feel strong, validated, and proud. Please get a screenshot ready before you start the survey.	¡Gracias! Al participar en el proyecto ¡SUBÉ DE NIVEL!, ¡estás ayudándonos a hacer que los espacios de videojuegos sean más abiertos para las juventudes LGBTQ+!<br><br>**Esta encuesta tardará aproximadamente 20 minutos en completarse.**<br><br>Por participar en esta investigación, nos gustaría darte las gracias al ofrecerte una oportunidad para participar en una rifa para una tarjeta de regalo con valor de $25 dólares canadienses para una tienda de videojuegos de tu elección (PlayStation, Xbox, Nintendo Switch, Steam, o iTunes). Más información estará disponible después de que entregues la encuesta.<br><br>En la encuesta, tendrás la oportunidad de subir una captura de pantalla de un videojuego que te haga sentir fuerte, validado y orgulloso. Por favor, prepare una captura de pantalla antes de comenzar la encuesta.
**1**	How old are you? [INSERT NUMBER]	¿Qué edad tienes? [RESPUESTAS DESPLEGABLES]
**2**	What country do you live in?<br>a) Australia<br>b) Canada<br>c) Mexico<br>d) United Kingdom<br>e) United States<br>f) Other (Please specify)	¿En qué país vives?<br>a) Australia<br>b) Canadá<br>c) México<br>d) Reino Unido<br>e) Estados Unidos<br>f) Otro (Escribe tu respuesta.)
**3**	Were you born in the country you are currently living in?<br>a) Yes<br>b) No	¿Naciste en el país en el que vives actualmente?<br>a) Sí<br>b) No

	**[ONLY APPEARS IF "NO" WAS THE RESPONSE TO THE PREVIOUS QUESTION]**<br><br>What country were you born in? [WRITE RESPONSE]<br><br>What age were you when you moved to the country you are living in now? [WRITE NUMERICAL RESPONSE]	**[ÚNICAMENTE APARECE SI LA PREGUNTA PREVIA, TUVO "NO" POR RESPUESTA.]**<br><br>¿En qué país naciste? [ESCRIBE RESPUESTA]<br><br>¿A qué edad te mudaste al país donde ahora vives? [ESCRIBE RESPUESTA EN FORMATO DE NÚMERO]
**4**	Which describe your gender identity/expression? Please check all that apply.<br>a) Agender<br>b) Cis Man/Cis Male<br>c) Cis Woman/Cis Female<br>d) Intersex<br>e) Man/Male of trans experience [FOR THE US AND CANADA ONLY]<br>f) Woman/Female of trans experience [FOR THE US AND CANADA ONLY]<br>g) Nonbinary<br>h) Gender Fluid<br>i) Gender Queer<br>j) Trans Man/Trans Masc/Trans Masculine<br>k) Trans Woman/Trans Female/Trans Feminine<br>l) Two-Spirit [US AND CANADA ONLY]<br>m) Muxe [MEXICO ONLY]<br>n) Brotherboy or Sistergirl [AUSTRALIA ONLY]<br>o) I identify as... [WRITE RESPONSE]<br><br>**[ONLY APPEARS IF RESPONSE TO THE PREVIOUS QUESTION INCLUDED MULTIPLE RESPONSES]**<br><br>Which describes your **most important** gender identity/expression?<br>a) Agender	¿Cuál describe tu expresión/identidad de género? Elige todas las que aplican.<br>a) Sin género<br>b) Hombre Cis<br>c) Mujer Cis<br>d) Persona intersexual<br>e) Hombre trans/Persona de experiencia transmasculina [SOLAMENTE ESTADOS UNIOS y CANADÁ]<br>f) Mujer/Persona de experiencia transfemenina [SOLAMENTE ESTADOS UNIDOS y CANADÁ]<br>g) No-binarie<br>h) de género fluido<br>i) de género cuir<br>j) Transmasculino<br>k) Transfemenina<br>l) Two-Spirit [SOLAMENTE ESTADOS UNIDOS y CANADÁ]<br>m) Muxe [SOLAMENTE MÉXICO]<br>n) Brotherboy o Sistergirl [AUSTRALIA ONLY]<br>o) Me identifico como... [ESCRIBE TU RESPUESTA]<br><br>**[SÓLO APARECE SI LA RESPUESTA A LA PREGUNTA ANTERIOR INCLUÍA MÚLTIPLES RESPUESTAS]**<br><br>¿Cuál describe tu expresión/identidad de género **más importantes**?<br>a) Sin género

	b) Cis Man/Cis Male<br>c) Cis Woman/Cis Female<br>d) Intersex<br>e) Man/Male of trans experience [FOR THE US AND CANADA ONLY]<br>f) Woman/Female of trans experience [FOR THE US AND CANADA ONLY]<br>g) Nonbinary<br>h) Gender Fluid<br>i) Gender Queer<br>j) Trans Man/Trans Masc/Trans Masculine<br>k) Trans Woman/Trans Female/Trans Feminine<br>l) Two-Spirit [US AND CANADA ONLY]<br>m) Muxe [MEXICO ONLY]<br>n) Brotherboy or Sistergirl [AUSTRALIA ONLY]<br>o) [RESPONSE FROM "O"]	b) Hombre Cis<br>c) Mujer Cis<br>d) Persona intersexual<br>e) Hombre trans/Persona de experiencia transmasculina [SOLAMENTE ESTADOS UNIDOS y CANADÁ]<br>f) Mujer/Persona de experiencia transfemenina [SOLAMENTE ESTADOS UNIDOS y CANADÁ]<br>g) No-binarie<br>h) de género fluido<br>i) de género cuir<br>j) Transmasculino<br>k) Transfemenina<br>l) Two-Spirit [SOLAMENTE ESTADOS UNIDOS y CANADÁ]<br>m) Muxe [SOLAMENTE MÉXICO]<br>n) Brotherboy o Sistergirl [SOLAMENTE AUSTRALIA]<br>o) [RESPUESTA DE "O"]
**5**	Which describe your sexual orientation? Please check all that apply.<br>a) Asexual<br>b) Aromantic<br>c) Bisexual/Biromantic<br>d) Demisexual/Demiromantic<br>e) Gay<br>f) Lesbian<br>g) Not sure or questioning<br>h) Pansexual/Panromantic<br>i) Queer<br>j) Straight/Heterosexual<br>k) I identify as... [WRITE IN RESPONSE]<br><br>**[ONLY APPEARS IF RESPONSE TO THE PREVIOUS QUESTION INCLUDED MULTIPLE RESPONSES]**	¿Cuál de estos describe tu orientación sexual? Elige todas las que applican.<br>a) Asexual<br>b) Aromántica<br>c) Bisexual/Birománticx<br>d) Demi/Demiromantix<br>e) Gay<br>f) Lesbiana<br>g) No estoy segurx/cuestionando<br>h) Pansexual/Panrománticx<br>i) Cuir<br>j) Heterosexual<br>k) Me identifico como… [ESCRIBE TU RESPUESTA]<br><br>**[SÓLO APARECE SI LA RESPUESTA A LA PREGUNTA ANTERIOR INCLUÍA MÚLTIPLES RESPUESTAS]**

	Which describes your **most dominant** sexual orientation?<br>a) Asexual<br>b) Aromantic<br>c) Bisexual/Biromantic<br>d) Demisexual/Demiromantic<br>e) Gay<br>f) Lesbian<br>g) Not sure or questioning<br>h) Pansexual/Panromantic<br>i) Queer<br>j) Straight/Heterosexual<br>k) [RESPONSE FROM K]	¿Cuál describe tu orientación **más dominante**?<br>a) Asexual<br>b) Arománticx<br>c) Bisexual/Birománticx<br>d) Demi/Demiromántix<br>e) Gay<br>f) Lesbiana<br>g) No estoy segurx/cuestionando<br>h) Pansexual/Panrománticx<br>i) Cuir<br>j) Heterosexual<br>k) [RESPUESTA DE "K"]
**8**	I am out to (have told people I am LGBTQ+)...<br>a) Everyone<br>b) Some people<br>c) No one - I am not out.<br><br>**[ONLY APPEARS IF RESPONSE TO THE PREVIOUS QUESTION WAS "A" OR "B"]**	¿Has salido del clóset (Le has dicho a otras personas que eres LGBTQ+)?<br>a) A todxs<br>b) Algunas personas<br>c) Nadie - No he salido<br><br>**[ÚNICAMENTE APARECE SI LA PREGUNTA PREVIA, TUVO "A" O "B" POR RESPUESTA.]**<br><br>¿Has salido del clóset (Le has dicho a otras personas que eres LGBTQ+)?<br>a) Algunas amistades (En línea)<br>b) Varias amistades (En línea)<br>c) Algunas amistades (Presencial)<br>d) Varias amistades (Presencial)<br>e) Una figura materna/paterna (incluyendo padres adoptivos, padres sustitutos, padrastro/madrastra, cuidadorxs)<br>f) Dos o más figuras maternas/paternas (incluyendo padres adoptivos, padres sustitutos, padrastro/madrastra, cuidadorxs)<br>g) Hermanos, hermanas, hermanxs<br>h) Parientes lejanos, primxs, tías, tíos, otros parientes

	Who are you out to (who have you told that you are LGBTQ+)?<br>a) A few friends (online)<br>b) Many friends (online)<br>c) A few friends (offline)<br>d) Many friends (offline)<br>e) One parent (including adopted parents, foster parents, stepparents, caregivers)<br>f) Two or more parents (including adopted parents, foster parents, stepparents, caregivers)<br>g) Sisters, brothers, siblings<br>h) Extended family, cousings, aunts, uncles, other relatives<br>i) A few classmates/Other students<br>j) Many classmates/Other students<br>k) Coworkers/Bosses<br>l) Teachers/School administration<br>m) Doctors/Health professionals<br>n) Other (Please specify)	i) Algunos compañerxs de clases/otrxs estudiantes<br>j) Varias compañerxs de clases/otrxs estudiantes<br>k) Colegas/Jefes o superiores<br>l) Maestrxs/Profesorxs/Administración escolar<br>m) Doctorxs/Profesionalxs de la salud<br>n) Otrx (por favor especifica)

**9**	Which of the following describe your racial/ethnic identity? Please select all that apply. [THIS QUESTION IS FOR CANADA, US, & UK ONLY]<br>a) Asian (e.g., South Asian, East Asian, etc.)<br>b) Black (e.g., Caribbean, African, etc.)<br>c) First Nations/Indigenous/Inuit/Metis<br>d) Hispanic/Latino/Latina/Latinx<br>e) Middle Eastern (e.g., Arab, etc.)<br>f) White (European, etc.)<br>g) Mixed/Multi-ethnic/Racial identity (Please specify) [WRITE IN RESPONSE]<br>h) None of these options describe my racial/ethnic identity. I identify as… [WRITE IN RESPONSE]<br><br>Which describes your ethnicity?<br>a) English, Welsh, Scottish, Northern Irish or British<br>b) Irish<br>c) Gypsy or Irish Traveller<br>d) Any other White background<br>e) White and Black Caribbean<br>f) White and Black African<br>g) White and Asian<br>h) Any other Mixed or Multiple ethnic background<br>i) Indian<br>j) Pakistani<br>k) Bangladeshi<br>l) Chinese<br>m) Any other Asian background<br>n) African<br>o) Caribbean<br>p) Any other Black, African, or Caribbean background<br>q) Arab<br>r) Any other ethnic group	¿Cuál describe tu identidad étnica/racial? (Elige todas las que aplican.) [ESTE PREGUNTA SOLAMENTE PARA ESTADOS UNIDOS, CANADÁ, y REINO UNIDO]<br>a) Asiáticx (ej. Asia del sur, Asia del oeste, etc.)<br>b) Negrx (ej. Caribeñx, Africanx, etc.)<br>c) First Nations/Indígenx/Inuit/Metis<br>d) Latino/Latina/Latinx<br>e) Del Medio Oriente (ej. árabe, libanés, etc.)<br>f) Caucásicx/blancx (europex, etc.)<br>g) Mestizx/Multi-étnicx/Identidad racial (Por favor especifica) [ESCRIBE TU RESPUESTA]<br>h) Ninguna de estas opciones describen mi etnicidad/identidad racial. Me identifico como... [ESCRIBE TU RESPUESTA]<br><br>¿Cuál de estos describe tu etnicidad?<br>a) Inglesx, galesx, escocesx, del norte de Irlanda o británicx<br>b) Irlandesx<br>c) Gitanx o Irish Traveller<br>d) Cualquier otro grupo de etnicidad blanca<br>e) Caribeñx blancx o negrx<br>f) Africanx blancx o negrx<br>g) Blancx y asiáticx<br>h) Cualquier otro grupo de etnicidad múltiple o mezclada<br>i) Indix<br>j) Pakistaní<br>k) Bangladeshi<br>l) Chinx<br>m) Cualquier otra etnicidad asiática<br>n) Africanx<br>o) Caribeñx<br>p) Cualquier otra etnicidad negra, africana o caribeña<br>q) Árabe<br>r) Cualquier otro grupo étnico

**10**	Do you have a disability?<br>a) Yes, and I have a formal diagnosis.<br>b) Yes, but I do not have a formal diagnosis<br>c) No<br>d) Prefer not to answer<br><br>**[ONLY APPEARS IF RESPONSE TO THE PREVIOUS QUESTION WAS "A" OR "B"]**<br><br>Your disability falls under which category/categories? Please check all that apply.<br>a) Vision (e.g., due to blindness or partial sight)<br>b) Hearing (e.g., due to deafness or partial hearing)<br>c) Mobility, such as difficulty walking short distances, climbing stairs, lifting and carrying objects<br>d) Head injury, stroke, or other acquired brain injury (e.g., traumatic brain injury)<br>e) Mental health condition<br>f) Stamina or breathing difficulty<br>g) Social, attention, or behavioural issues (e.g., Attention Deficit Hyperactivity Disorder)<br>h) Autism Spectrum Disorder<br>i) Learning (e.g., learning disability, dyslexia, dyscalculia)<br>j) Communication (e.g., speech difficulties or language disorders)<br>k) Other (Please specify) [WRITE IN RESPONSE]	¿Te han diagnosticado o te han clasificado con una discapacidad?<br>a) Sí, y tengo una diagnostico formál<br>b) Sí, pero no tengo una diagnostico formál<br>c) No<br>d) Prefiero no responder<br><br>**[ÚNICAMENTE APARECE SI LA PREGUNTA PREVIA, TUVO "A" O "B" POR RESPUESTA.]**<br><br>¿Su discapacidad cae bajo qué categoría/categorías? Por favor, marque todo lo que corresponda.<br>a) Visión (por ejemplo, debido a ceguera o visión parcial)<br>b) Audición (por ejemplo, debido a sordera o audición parcial)<br>c) Movilidad, como dificultad para caminar distancias cortas, subir escaleras, levantar y transportar objetos<br>d) Lesión en la cabeza, accidente cerebrovascular u otra lesión cerebral adquirida (por ejemplo, lesión cerebral traumática)<br>e) Condición de salud mental<br>f) Resistencia o dificultad para respirar<br>g) Problemas sociales, de atención o de comportamiento (por ejemplo, trastorno por déficit de atención con hiperactividad)<br>h) Trastorno del espectro autista<br>i) Aprendizaje (por ejemplo, discapacidad de aprendizaje, dislexia, discalculia)<br>j) Comunicación (por ejemplo, dificultades del habla o trastornos del lenguaje)<br>k) Otros (especifique)[ESCRIBE TU RESPUESTA]

	SECTION 2: VIDEO GAME PLAYSTYLE & PREFERENCES	SECCIÓN 2: ESTILO DE JUEGO DE VIDEOJUEGO Y PREFERENCIAS
**11**	What age were you when you started playing video games regularly? [INSERT NUMBER]	¿Qué edad tenías cuando comenzaste a jugar videojuegos de forma regular? [RESPUESTAS DESPLEGABLES]
**12**	In a typical week, how much time do you spend (in hours) playing video games? Please enter a number.	Durante una semana típica, ¿cuánto tiempo pasas jugando videojuegos? Inserta un número.
**13**	If you didn't have any commitments (e.g., work, school, etc.) how much time (in hours) would you prefer to spend playing video games in a week? Please enter a number.	Si no tuvieras compromisos (ej., trabajo, escuela, etc.) ¿cuánto tiempo preferirías pasar jugando videojuegos en una semana? Inserta un número.
**14**	Which of the following do you use to play video games? Please select all that apply.<br>a) Nintendo (i.e., Nintendo Switch, WiiU, Nintendo 3DS)<br>b) PlayStation (i.e., PlayStation 5, PlayStation 4, PS Vita)<br>c) Xbox (i.e., Xbox Series X, Xbox One, Xbox 360)<br>d) Virtual Reality (i.e., Oculus Rift, HTC Vive, PlayStation VR)<br>e) PC or laptop<br>f) Tablet or smartphone<br>g) Other (Please specify) [WRITE IN RESPONSE]	¿Cuál de los siguientes usas para jugar juegos? (Selecciona todos los que apliquen.)<br>a) Nintendo (ej., Nintendo Switch, WiiU, Nintendo 3DS)<br>b) PlayStation (ej., PlayStation 5, PlayStation 4, PS Vita)<br>c) Xbox (ej., Xbox Series X, Xbox One, Xbox 360)<br>d) Virtual Reality (i.e., Oculus Rift, HTC Vive, PlayStation VR)<br>e) PC o laptop<br>f) Tableta o teléfono inteligente<br>g) Otro (Por favor especifica) [ESCRIBE TU RESPUESTA]

**15**	Why do you play video games?<br>a) For fun and entertainment<br>b) To reduce stress<br>c) To pass time<br>d) To connect with friends<br>e) To meet new people<br>f) To compete for ranks, titles, etc.<br>g) To level up my skills and unlock more gameplay<br>h) To immerse myself in a story<br>i) To express my creativity and experiment in-game<br>j) To master challenging gameplay (e.g., hard difficulties, speed-runs)<br>k) To complete things as fully as possible (e.g., gathering in-game collectibles, earning all trophies)<br>l) Other (Please specify) [WRITE IN RESPONSE]	¿Cuándo juegas videojuegos? Selecciona todos los que apliquen.<br>a) Por diversión y entretenimiento<br>b) Para reducir el estrés<br>c) Nada más para pasar el tiempo<br>d) Para conectarse con amigxs<br>e) Para conocer personas nuevas<br>f) Para competir y ganar títulos, rangos, etc.<br>g) Para mejorar mis habilidades y desbloquear más del juego<br>h) Para involucrarme en una historia<br>i) Para expresar mi creatividad y experimentar más en el juego<br>j) Para hacerme mejor jugador y superar los desafíos (ej., dificultades elevadas, speed-runs)<br>k) Para completar las cosas lo más pronto posible (ej., conseguir los coleccionables dentro de los juegos, conseguir todos los trofeos)<br>l) Otro (Opción para especificar.) [ESCRIBE TU RESPUESTA]
**17**	Do you play… [OPTIONS: never, sometimes, often, always]<br><br>**<u>SINGLE PLAYER GAMES</u>**<br>**single player games** by yourself?<br><br>**single player games** with people you know well (i.e., close friends, family)? For example, playing a single player game with others watching or passing the controller back and forth to take turns.<br><br>**single player games** with people you do not know very well (i.e., acquaintances, strangers)? For example, in a college dorm room playing a single player game with others watching or passing the controller back and forth to take turns.	Cuando juegas, son...¿ [OPCIONES: nunca, a veces, seguido, casi siempre]<br><br>**<u>JUEGO DE JUGADOR</u>**<br>**juegos de jugadorxs** solitarixs**?**<br><br>**juegos de jugadorxs solitarixs** con personas a las que conoces bien? (ej., amigxs cercanos, familia)? Pase el cursor sobre esta pregunta para ver un ejemplo.<br><br>**juegos de jugadorxs solitarixs** con personas a las que no conoces bien? (ej., conocidxs, extrañxs)? Pase el cursor sobre esta pregunta para ver un ejemplo.

	**<u>MULTIPLAYER GAMES</u>**<br>**multiplayer games** with people you know well (i.e., close friends, family)?<br><br>**multiplayer games** with people you do not know very well (i.e., acquaintances, strangers)?<br><br>**<u>ONLINE COMMUNITIES</u>**<br>**games while streaming** to an audience (i.e., Twitch, YouTube)?	**<u>JUEGOS CON MÚLTIPLES JUGADORXS</u>**<br>**juegos con múltiples jugadorxs** con personas a las que conoces bien? with people you know well (ej., amigxs cercanos, familia)?<br><br>**juegos con múltiples jugadorxs** con personas a las que no conoces bien? (ej., conocidxs, extrañxs)?<br><br>**<u>COMMUNIDADES EN LINEA</u>**<br>**juegos al hacer streaming** para una audiencia (ej. Twitch, YouTube)
**19**	Do you use or visit online video game communities and spaces (i.e., Twitch, video game-related message boards, Discord communities, etc.)?<br>a) Yes<br>b) No	¿Usas o visitas comunidades y espacios de videojuegos en línea (por ejemplo Twitch, mensajes y foros, comunidades de Discord, etc.)?<br>a) Sï<br>b) No

**20**	What are your favourite video game genres to play? Please select all that apply. *Note, many video games can fall under multiple categories so you can select as many as you wish. For example, the Last of Us can be considered both "Action" and "Horror."*<br><br>**Action** (e.g., Far Cry 6, Monster Hunter, Super Mario 3D World, Super Mario Odyssey, Red Dead Redemption II, God of War, la serie de The Last of Us)<br><br>**Adventure** (e.g., The Legend of Zelda series, Sekiro: Shadows Die Twice, Assassin's Creed series, Escape from Monkey Island, Star Wars: Fallen Order, Monster Hunter)<br><br>**Arcade** (e.g., Tetris Effect, Toree 2, Cooking Simulator, Pacman 99, Crossy Road)<br><br>**Battle Royale** (e.g., Fortnite, PUBG, Warzone, Apex Legends)<br><br>**Card Games** (e.g., Hearthstone, Gwent, Slay the Spire, Armello)<br><br>**Casual** (e.g., Candy Crush, Adventure Capitalist, Merge Mayor, Dragon's Den Tycoon, Pancake – The Game<br><br>**Fighting** (e.g., Mortal Kombat, Street Fighter, Super Smash Bros)<br><br>**First & Third Person Shooter** (e.g., First Person: Call of Duty, Star Wars Battlefront, Battlefield, Destiny, Titanfall, Valorant, Overwatch; Third Person: Gears of War, Metalgear Solid series, Warframe)<br><br>**Gacha** (e.g., Brave Frontier, Dragalia Lost, Final Fantasy Brave Exvius, Genshin Impact, Mario Kart World Tour, Monster Strike, Dragon Ball Z: Dokkan Battle, Identity V)<br><br>**Horror/Survival Horror** (e.g., The Last of Us series, Dying Light, Resident Evil, Dark Pictures Anthology, Fatal Frame o Silent Hill,	¿Cuáles son tus géneros preferidos de videojuegos al jugar? Selecciona todos los que apliquen. *Ojo; muchos videojuegos pueden caer bajo varias categorías, entonces selecciona todas las que gustes. Por ejemplo, the Last of Us se puede considerar Acción y Horror.*<br><br>**Acción** (ej., Far Cry 6, Monster Hunter, Super Mario 3D World, Super Mario Odyssey, Red Dead Redemption II, God of War, la serie de The Last of Us)<br><br>**Aventura** (ej., la serie de The Legend of Zelda, Sekiro: Shadows Die Twice, la serie de Assassin's Creed, Escape from Monkey Island, Star Wars: Fallen Order, Monster Hunter)<br><br>**Arcade** (ej., Tetris Effect, Toree 2, Cooking Simulator, Pacman 99, Crossy Road)<br><br>**Battle Royale** (ej., Fortnite, PUBG, Warzone, Apex Legends)<br><br>**Juegos de cartas** (e.g., Hearthstone, Gwent, Slay the Spire, Armello)<br><br>**Casual** (ej., Candy Crush, Adventure Capitalist, Merge Mayor, Dragon's Den Tycoon, Pancake – The Game)<br><br>**Peleas** (ej., Mortal Kombat, Street Fighter, Super Smash Bros)<br><br>**Tirador en primera y tercera persona** (ej., Primera persona: Call of Duty, Star Wars Battlefront, Battlefield, Destiny, Titanfall, Valorant, Overwatch; Tercera persona: Gears of War, Metalgear Solid series, Warframe)<br><br>**Gacha** (ej., Brave Frontier, Dragalia Lost, Final Fantasy Brave Exvius, Genshin Impact, Mario Kart World Tour, Monster Strike, Dragon Ball Z: Dokkan Battle, Identity V)<br><br>**Horror/Horror y supervivencia** (ej., series como The Last of Us, Dying Light, Resident Evil, Dark Pictures Anthology, Fatal Frame o Silent Hill,

	Senua's Sacrifice, Death Stranding, Siren: Blood Curse, Dead by Daylight)<br><br>**Music/Rhythm** (e.g., Guitar Hero, Rock Band, Beat Saber, Cadence of Hyrule)<br><br>**Platformer** (e.g., Cuphead, Metroid: Dread, Super Mario All Stars, Super Mario World, Celeste, Sonic the Hedgehog)<br><br>**Puzzle** (e.g., Portal 2, Tetris, Dr. Mario)<br><br>**Party Play** (e.g., Jackbox Party Pack, Mario Party, Overcooked, Among Us, Mario Kart)<br><br>**Racing** (e.g., Mario Kart, Real Racing 3, Need for Speed Heat, GRID™ Autosport)<br><br>**Role-Playing Game** (e.g., Final Fantasy series, Earthbound, Shin Megami Tensei series, Persona series, Neo: The World Ends With You, Skyrim, The Witcher 3, Fallout 4)<br><br>**Sandbox** (e.g., Minecraft, Grand Theft Auto, The Sims, Watchdogs, LEGO Worlds)<br><br>**Strategy** (e.g., Warcraft, Age of Empires, Command & Conquer)<br><br>**Sports** (e.g., Madden NFL, NBA 2K, Forza Motorsport)<br><br>**MMO** (Massively Multiplayer Online; e.g., Final Fantasy XIV Online, World of Warcraft, EVE Online, Elder Scrolls Online)<br><br>**MOBA** (Multiplayer Online Battle Arena; e.g., League of Legends, Dota series, Heroes of the Storm, Smite)<br><br>**Mobile** (e.g., Pokémon GO, Candy Crush)<br><br>**Virtual Reality** (e.g., Superhot, Lucky's Tale)<br><br>**Other:** Please specify	Senua's Sacrifice, Death Stranding, Siren: Blood Curse, Dead by Daylight)<br><br>**Música/ritmo** (ej., Guitar Hero, Rock Band, Beat Saber, Cadence of Hyrule)<br><br>**Plataforma** (ej., Cuphead, Metroid: Dread, Super Mario All Stars, Super Mario World, Celeste, la serie de Sonic the Hedgehog)<br><br>**Puzzle/Rompecabezas** (ej., Portal 2, Tetris, Dr. Mario)<br><br>**Party Play** (ej., Jackbox Party Pack, series como Mario Party, Overcooked, Among Us, Mario Kart)<br><br>**Carreras** (ej., Mario Kart, Real Racing 3, Need for Speed Heat, GRID™ Autosport)<br><br>**Juegos de rol** (ej., la serie de Final Fantasy, Earthbound, la serie de Shin Megami Tensei, la serie de Persona, Neo: The World Ends With You, Skyrim, The Witcher 3, Fallout 4)<br><br>**Sandbox** (ej., Minecraft, Grand Theft Auto, The Sims, Watchdogs, LEGO Worlds)<br><br>**Estrategia** (ej., Warcraft, Age of Empires, Command & Conquer)<br><br>**Deportes** (ej., Madden NFL, NBA 2K, Forza Motorsport)<br><br>**MMO** (Multijugador masivo en línea; e.g., Final Fantasy XIV Online, World of Warcraft, EVE Online, Elder Scrolls Online)<br><br>**MOBA** (Multijugador en línea del tipo Battle Arena; ej., League of Legends, Dota series, Heroes of the Storm, Smite)<br><br>**Móvil** (ej., Pokémon GO, Candy Crush)<br><br>**Realidad virtual** (ej., Superhot, Lucky's Tale)<br><br>**Otro:** Por favor especifica

	**SECTION 3.1: EXPERIENCES WITH VIDEO GAME CONTENT**<br><br>**[SECTION ONLY APPEARS IF RESPONSE TO SINGLE PLAYER PROMPTS IN SECTION 2, QUESTION 17 WAS NOT "NEVER"]**<br><br>**Instructions:** The following questions will help us learn more about the experiences you have had involving the content of the video games you play.	**SECCIÓN 3.1: EXPERIENCIES CON CONTENIDO DE VIDEOJUEGOS**<br><br>**[LA SECCIÓN SÓLO APARECE SI LA RESPUESTA A LAS INDICACIONES DE UN SOLO JUGADOR EN LA SECCIÓN 2, PREGUNTA 17 NO FUE "NUNCA"]**<br><br>**Instrucciones:** Las siguientes preguntas nos ayudarán a aprender mas sobre las experiencias que has tenido en relación con ele contenido de los videojuegos a los que juegas.
**20**	Have you identified with or related to any individual video game characters?<br>a) Yes<br>b) No<br><br>**[ONLY APPEARS IF RESPONSE TO THE PREVIOUS QUESTION WAS "A"]**<br><br>(21a) Name a video game character that you have identified with or related to and briefly describe how you identified with or related to them. (e.g., I relate to Dorian from Dragon Age: Inquisition because we both…) [WRITE IN RESPONSE]	¿Te has identificado o relacionado con algún personaje individual de un videojuego?<br>a) Sí<br>b) No<br><br>**[ÚNICAMENTE APARECE SI LA PREGUNTA PREVIA, TUVO "A" POR RESPUESTA.]**<br><br>(21a) Nombra a un personaje de videojuego con el que te hayas identificado o con el que te hayas relacionado y describe brevemente cómo te identificaste con él o con el que te relacionaste con él. (por ejemplo, me relaciono con Dorian de Dragon Age: Inquisition porque los dos...) [ESCRIBE TU RESPUESTA]
**22**	Upload a screenshot from a video game that makes you feel strong, validated, and proud. [Link here to instructions]<br><br>**[ONLY APPEARS IF UPLOADED A CHARACTER]**<br><br>Why did you select the image that you have just uploaded?	Sube un capture de pantalla del videojuego que te haga sentir fuerte, validado y orgulloso. [Enlace aquí a las instrucciones]<br><br>**[SOLO APARECE SI SE HA SUBIDO UN CARÁCTER]**<br><br>¿Por qué seleccionaste la imagen que acabas de subir?

**23**	Have you identified with or related to any video game storylines?<br>a) Yes<br>b) No<br><br>**[ONLY APPEARS IF RESPONSE TO THE PREVIOUS QUESTION WAS "YES"]**<br><br>(23a) Please briefly describe a video game storyline that you identified with or related to and describe why you identified with or related to it. (i.e., In *Life is Strange 2*, I identified with the storyline where Sean imagines a conversation about coming out to his father because...) [WRITE IN RESPONSE]	¿Te has identificado o relacionado con alguna trama de videojuego?<br>a) Sí<br>b) No<br><br>**[SÓLO APARECE SI LA RESPUESTA A LA PREGUNTA PREVIA FUE "SÍ"]**<br><br>(23a) Describa brevemente una historia de videojuego con la que se identificó o con la que se relacionó y describa por qué se identificó con ella o se relacionó con ella. (ej., En *Life is Strange 2*, me identifiqué con la historia donde Sean imagina una conversación sobre salir del clóset con su padre porque...) [ESCRIBE TU RESPUESTA]
**24**	Has it been important for you to identify with video game characters or storylines?<br>a) Yes<br>b) No<br><br>**[ONLY APPEARS IF RESPONSE TO THE PREVIOUS QUESTION WAS "YES"]**<br><br>(24a) Briefly describe why identifying with video game characters or storylines has been important to you. (e.g., I find this important because…)	¿Ha sido importante para tí identificarte con personajes o tramas de videojuegos?<br>a) Sí<br>b) No<br><br>**[SÓLO APARECE SI LA RESPUESTA A LA PREGUNTA PREVIA FUE "SÍ"]**<br><br>(24a) ¿Por qué ha sido importante para tí esta identificación? (ej., Considero que es importante por que…)

<table>
<tr><td>25</td><td>Instructions: The following questions help us get to know more about the experiences you have had involving LGBTQ+ content in the video games you play.

I have seen LGBTQ+ people represented in video game characters.
a) Yes
b) No

[ONLY APPEARS IF RESPONSE TO THE PRIOR QUESTION WAS “YES”]

(25a) In general, I find LGBTQ+ representation in video game characters to be...
a) Completely positive
b) Mostly positive
c) Neutral
d) Mostly negative
e) Completely negative</td><td>Instrucciones: Las siguientes preguntas nos ayudarán a aprender más acerca de las experiencias que has tenido con contenidos LGBTQ+ en los videojuegos que juegas.

He visto personas LGBTQ+ representadas en videojuegos.
a) Sí
b) No

[SÓLO APARECE SI LA RESPUESTA A LA PREGUNTA PREVIA FUE "SÍ"]

(25a) En general, encuentro que la representación LGBTQ + en personajes de videojuegos es ...
1. Completamente positivo
2. Mayormente positivo
3. Neutral
4. Mayormente negativo
5. Completamente negativo</td></tr>
<tr><td>26</td><td>I have experienced messages about LGBTQ+ themes in video game storylines.
a) Yes
b) No

[ONLY APPEARS IF RESPONSE TO THE PRIOR QUESTION WAS “YES”]

(27) In general, I find LGBTQ+ representation in video game storylines to be...
a) Completely positive
b) Mostly positive
c) Neutral
d) Mostly negative
e) Completely negative</td><td>He experimentado mensajes sobre o visto personas LGBTQ+ representadxs en historias en videojuegos.
a) Sí
b) No

[SÓLO APARECE SI LA RESPUESTA A LA PREGUNTA PREVIA FUE "SÍ"]

(27) En general, encuentro que la representación LGBTQ + en las historias de los videojuegos es ...
1. Completamente positivo
2. Mayormente positivo
3. Neutral
4. Mayormente negativo
5. Completamente negativo</td></tr>
</table>

**28**	I have used video game character creators or character selection tools (i.e., tools that allow you to customize or create characters in a video game or to select a character).<br>a) Yes<br>b) No<br><br>**[ONLY APPEARS IF RESPONSE TO THE PRIOR QUESTION WAS "YES"]**<br><br>I have been able to select video game characters I am satisfied with.<br>a) Yes<br>b) No	He usado creadores de personajes en videojuegos o herramientas para seleccionar los personajes (Ejemplos: herramientas que te permiten personalizar o crear personajes en un videojuego o seleccionar personajes).<br>a) Sí<br>b) No<br><br>**[SÓLO APARECE SI LA RESPUESTA A LA PREGUNTA PREVIA FUE "SÍ"]**<br><br>He podido crear o seleccionar personajes con los que estoy satisfechx.<br>a) Sí<br>b) No
**29**	I want to create or select video game characters that represent me.<br>a) Yes<br>b) No	Quiero crear o seleccionar personajes que me representan.<br>a) Sí<br>b) No
**30**	What type of features are you **satisfied** with in the characters that you've been able to create or select? [WRITE IN RESPONSE]	¿Qué tipo de características o distintivas te hacen sentir **satisfechx** con los personajes que has creado o seleccionado? [ESCRIBE TU RESPUESTA]
**31**	What types of features are you **dissatisfied** with in the characters that you've been able to create or select? [WRITE IN RESPONSE]	¿Qué tipo de características o distintivas te hacen sentir **insatisfechx** con los personajes que has podido crear o seleccionar? [ESCRIBE TU RESPUESTA]
**32**	**Optional:** Is there anything else you would like to tell us about your experiences with LGBTQ+ content you have seen in video games? [WRITE IN RESPONSE]	**Opcional:** ¿Hay algo más que quisieras contarnos acerca de tus experiencias con los contenidos LGBTQ+ que has visto en videojuegos? [ESCRIBE TU RESPUESTA]

	SECTION 3.2: IMPACT OF NEGATIVE EXPERIENCES WITH VIDEO GAME CONTENT	SECCIÓN 3.2: IMPACTO DE LAS EXPERIENCIAS NEGATIVAS CON EL CONTENIDO DE VIDEOJUEGO
**33**	Have you had a **negative** gaming experience involving video game characters or storylines that led you to think about your LGBTQ+ identity?<br>a) Yes<br>b) No [SKIP TO SECTION 3.3]	¿Has tenido una experiencia negativa jugando videojuegos que llevó que pensaras o te cuestionaras sobre tu identidad LGBTQ+?<br>a) Sí<br>b) No [SALTAR A SECCIÓN 3.3]
**34**	Briefly tell us about a **negative** experience involving video games characters or storylines that led you to think about your LGBTQ+ identity. [WRITE IN RESPONSE]	Cuéntanos sobre una experiencia negativa a la hora de jugar que te hizo pensar sobre tu identidad LGBTQ+. [ESCRIBE TU RESPUESTA]
**35**	**Instructions:** Thinking about all of the **negative** experiences with **video game content**, how much were you impacted in the following ways?<br><br>**RATING SCALE:** (0 – 4). [The default is at 0].<br>I was not affected this way at all; I was affected this way a little bit; I was somewhat affected this way; I was affected this way quite a bit; I was very much affected this way	**Instrucciones:** Piensa sobre la experiencia negativa que describiste arriba. Por favor indica qué tanto los siguientes enunciados describen el impacto durante o después de la experiencia que compartiste.<br><br>**ESCALA DE RANGO:** (0 – 4). [El default está en 0].<br>No me afectó en lo absoluto; me afectó de esta manera un poco; me afectó algo de esta manera; me afectó de esta manera bastante; me afectó mucho de esta manera.
	I felt angry.	Me sentí enojadx.
	I felt frustrated.	Me sentí frustradx.
	I felt upset.	Me sentí molestx.
	I was in shock.	Estaba en shock.
	I felt like I had no control.	Sentí que perdía control.
	I felt embarrassed.	Me sentí avergonzadx.
	I felt anxious.	Me sentí ansiosx.
	I felt worthless.	Me sentí inútil.

	I felt lost.	Me sentí perdidx.
	I stopped playing the game.	Dejé de jugar el juego.
	I blocked someone.	Bloqueé a alguien.
	I deleted/uninstalled the game.	Eliminé / desinstalé el juego.
	I played the game less.	Jugué menos al juego.
	I went to see a psychologist/counselor.	Fui a ver a un psicólogx / consejerx.
	I got (or tried to get) support from a friend or other trusted adult.	Recibí (o traté de obtener) el apoyo de un amigx u otro adultx de confianza.
	I felt worse about being an LGBTQ+ person.	Me sentí peor por ser una persona LGBTQ+.
	I felt better about being an LGBTQ+ person.	Me sentí mejor por ser una persona LGBTQ+.
	I was ***less*** likely to talk about being LGBTQ+ with people **ONline**.	Era ***menos*** probable que hablara sobre ser LGBTQ + con personas **en línea**.
	I was ***less*** likely to talk about being LGBTQ+ with people **OFFline**.	Era ***menos*** probable que hablara sobre ser LGBTQ + **en persona**.
	I was ***more*** likely to talk about being LGBTQ+ with people **ONline**.	Era ***más*** probable que hablara sobre ser LGBTQ + con personas **En línea**.
	I was ***more*** likely to talk about being LGBTQ+ with people **OFFline**.	Era ***más*** probable que hablara sobre ser LGBTQ + **en persona**.
	I feel like I have become a better person.	Siento que me he convertido en una mejor persona.
	I learned more about myself.	Aprendí más sobre mí mismo.
	It made me realise what was really important to me.	Me hizo darme cuenta de lo que era realmente importante para mí.
	I felt like a stronger person after the experience.	Me sentí como una persona más fuerte después de la experiencia.
	My experience made me more sensitive to others who have similar experiences.	Mi experiencia me hizo más sensible a otros que tienen experiencias similares.
**36**	**Optional:** Please share more about **the impacts** of any **negative** experiences with **video game content** you have had related to your LGBTQ+ identity. [WRITE IN RESPONSE]	**Opcional:** Por favor comparte más acerca de **los impactos** de cualquier experiencia negativa que has tenido relacionado con ser LGBTQ+ en los videojuegos. [ESCRIBE TU RESPUESTA]

	SECTION 3.3: IMPACT OF POSITIVE EXPERIENCES WITH VIDEO GAME CONTENT	SECCIÓN 3.3: IMPACTO DE LAS EXPERIENCIAS POSITIVAS CON EL CONTENIDO DE VIDEOJUEGOS
**37**	Have you had a **positive** gaming experience involving video game characters or storylines that led you to think about your LGBTQ+ identity?<br>c) Yes<br>d) No [SKIP TO SECTION 4.1]	¿Has tenido una experiencia positiva jugando videojuegos que llevó que pensaras o te cuestionaras sobre tu identidad LGBTQ+?<br>c) Sí<br>d) No [SALTAR A SECCIÓN 4.1]
**37a**	Briefly tell us about a **positive** experience involving video games characters or storylines that led you to think about your LGBTQ+ identity. [WRITE IN RESPONSE]	Cuéntanos sobre **una experiencia positiva** a la hora de jugar que te hizo pensar sobre tu identidad LGBTQ+. [ESCRIBE TU RESPUESTA]
**38**	**Instructions:** Thinking about all of the **positiva** experiences with **video game content**, how much were you impacted in the following ways?<br><br>**RATING SCALE:** (0 – 4). [The default is at 0].<br>I was not affected this way at all; I was affected this way a little bit; I was somewhat affected this way; I was affected this way quite a bit; I was very much affected this way	**Instrucciones:** Piensa sobre **la experiencia positiva** que describiste arriba. Por favor indica qué tanto los siguientes enunciados describen el impacto durante o después de la experiencia que compartiste.<br><br>**ESCALA DE RANGO:** (0 – 4). [El default está en 0].<br>No me afectó en lo absoluto; me afectó de esta manera un poco; me afectó algo de esta manera; me afectó de esta manera bastante; me afectó mucho de esta manera.
	I felt happy.	Me sentí feliz.
	I felt supported.	Me sentí apoyadx.
	I felt comfortable.	Me sentí cómodx.
	I felt like part of a group.	Me sentí como parte de un grupo.
	I felt less stress.	Sentí menos estrés.
	I felt less anxiety.	Sentí menos ansiedad.

	I played the game more.	Jugué más el juego.
	I added or friended someone.	Agregué a alguien como amigx.
	I played the game less.	Jugué menos el juego.
	I felt worse about being an LGBTQ+ person.	Me sentí peor por ser una persona LGBTQ+.
	I felt better about being an LGBTQ+ person.	Me sentí mejor por ser una persona LGBTQ+.
	I was ***less*** likely to talk about being LGBTQ+ with people **ONline**.	Estoy ***menos*** cómodx con hablar acerca de ser LGBTQ+ con personas **en línea**.
	I was ***less*** likely to talk about being LGBTQ+ with people **OFFline**.	Estoy ***menos*** cómodx con hablar acerca de ser LGBTQ+ **en persona**.
	I was ***more*** likely to talk about being LGBTQ+ with people **ONline**.	Estoy ***más*** cómodx con hablar acerca de ser LGBTQ+ con personas **en línea**.
	I was ***more*** likely to talk about being LGBTQ+ with people **OFFline**.	Estoy ***más*** cómodx con hablar acerca de ser LGBTQ+ **en persona**.
	I feel like I have become a better person.	Siento que me convertido en mejor persona.
	I learned more about myself.	Aprendí más acerca de mí mismx.
	It made me realize what was really important to me.	Me hizo darme cuenta de lo que realmente es importante para mí.
	I learned to stand up for myself.	Aprendí a defenderme.
	I learned not to care as much about what people think about me.	Aprendí a que me importara menos lo que la gente piensa de mí.
	I felt like a stronger person after the experience.	Me sentí como una persona más fuerte después de la experiencia.
	My experience made me more sensitive to others who have similar experiences.	Mi experiencia me hizo más sensible a otrxs que han tenido experiencias similares.
**39**	**Optional**: Please share more about **the impacts** of any **positive** experiences with **video game content** you have had related to your LGBTQ+ identity. [WRITE IN RESPONSE]	**Opcional**: Por favor compártenos más acerca de **los impactos** de cualquier experiencia positiva que has tenido relacionada con tu experiencia LGBTQ+. [ESCRIBE TU RESPUESTA]

	**SECTION 4.1: NEGATIVE EXPERIENCES IN MULTIPLAYER GAMES, GAMING COMMUNITIES/SPACES & STREAMS**	**SECCIÓN 4.1: EXPERIENCIAS NEGATIVAS JUGANDO JUEGOS DE JUGADORXS MÚLTIPLES, USANDO COMUNIDADES Y ESPACIOS DE JUEGO, Y O JUEGOS CON STREAMING**
	**[SECTION ONLY APPEARS IF RESPONSE TO MULTIPLAYER/GAMING COMMUNITY PROMPTS IN SECTION 2, QUESTION 17 WAS NOT "NEVER"]**	**[LA SECCIÓN SOLO APARECE SI LA RESPUESTAS AL MULTIJUGADOR/ COMUNIDAD DE JUEGOS EN LA SECCIÓN 2, PREGUNTA 17 NO FUE "NUNCA"]**
**40**	**EXPERIENCE (1/3)**<br><br>**Instructions**: For each of the following, please indicate whether the experience described has happened to you, happened to others, is a regular and accepted part of gaming culture, or has never happened while playing multiplayer games (including voice or text chat) or when using gaming communities and spaces (for example, Twitch, Discord, Reddit, etc.).<br><br>These questions will span multiple pages.<br><br>**Content warning:** The following items ask you to recall homophobic and/or transphobic instances that you may have experienced or observed while playing video games. There is mention of slurs that have been used to harm LGBTQ+ people. Please take a minute to take a break or take care of your wellbeing.<br><br>[OPTIONS: **this happened to me, I saw this happen to others, this feels like an accepted part of gaming culture, I haven't experienced or witnessed this**]	**EXPERIENCIA (1/3)**<br><br>**Instrucciones:** Para cada una de las siguientes, por favor indica si la experiencia descrita te sucedió a tí, le sucedió a otrxs, es una parte regular y aceptada de la cultura de los videojuegos o nunca ha pasado al jugar juegos con jugadorxs múltiples (incluyendo voz o chat por texto) o cuando se usan comunidades o espacios de juego (por ejemplo, Twitch, Discord, Reddit, etc.)<br><br>Estas preguntas abarcarán varias páginas.<br><br>**Advertencia de contenido:** Los siguientes elementos piden que recuerdes instancias homofóbicas y/o transfóbicas que pudiste haber experimentado u observado al jugar videojuegos. Hay mención de insultos que han sido usados para hacerle daño a las personas LGBTQ+. Por favor tómate un minuto para respirar o cuidar tu bienestar.<br><br>[OPCIONES: **esto me sucedió a mí, vi que esto le pasó a otrxs, esto se siente como una parte aceptada de la comunidad de los videojuegos, no he experimentado ni he sido testigo de esto.**]
	**Someone said or implied...**	**Alguien dijo (o implicó)...**

	being LGBTQ+ is a “just a phase.”	ser LGBTQ+ es “nada más una fase.”
	it is a choice to be LGBTQ+.	es una elección ser LGBTQ+.
	an anti-LGBTQ+ slur (e.g., “fag,” “queer”).	un insulto anti-LGBTQ+ (ej., “puto” “maricón).
	a negative LGBTQ+ joke.	un chiste contra lo LGBTQ+.
	that you/others were overly sensitive for thinking that you/they were being treated unfairly because of your or their LGBTQ+ identity.	que tú/otrxs eran súmamente sensiblxs por pensar que tú/ellxs estaban siendo tratadxs de una manera injusta por su/tu identidad LGBTQ+.
	the wrong personal pronouns or name to reference you/others.	los pronombres equivocados, o el nombre equivocado para hablar de tí o de otrxs.
	that an anti-LGBTQ+ comment didn’t mean anything, or it was just a joke.	que un comentario anti-LGBTQ+ no significaba nada o era solo un chiste.
	that you/others were overreacting in response to an anti-LGBTQ+ joke (e.g., called them a snowflake or over-sensitive).	que tú/otrxs estaban sobre-reaccionando en respuesta a un chiste anti-LGBTQ+ (que les llamaran sobre-sensible o ridículx).
	“that’s how the real world is” in a conversation about anti-LGBTQ+ prejudice and discrimination.	"así es el mundo” en una conversación sobre prejuicios y discriminación contra lo LGBTQ+
	that you/others should stop complaining about anti-LGBTQ+ prejudice and discrimination.	que tú/otrxs deberían dejar de quejarse sobre prejuicios y discriminación contra lo LGBTQ+
	that it is wrong to be LGBTQ+ or that you/others will go to hell because of your/others’ LGBTQ+ identity.	que está mal ser LGBTQ+ o que tú/otrxs se irán al infierno por la identidad LGBT que tienen
	that LGBTQ+ is a result of something that went “wrong” in your/others’ past (e.g., “mother was too overbearing,” “father wasn’t around”).	que ser LGBTQ+ es resultado de que algo “salió mal” en tu pasado o el de otrxs (ej., “tu mamá fue muy dominante”, o “tu padre no estuvo presente").
	that they were praying for you/others because they knew or assumed that you/others were LGBTQ+.	que estaban rezando por tí/otrxs porque saben o asumen que eres/son LGBTQ+.
	that you/others would hit on them because of your LGBTQ+ identity.	que tú/otrxs se insinuarían sexualmente a ellxs debido a su identidad LGBTQ+.

41	**SETTING (2/3)**<br><br>**Please indicate where the event(s) happened**<br><br>**[OPTIONS: playing a multiplayer game with close friends or family, playing a multiplayer game with acquaintances and/or strangers, in a gaming community (i.e., Twitch, Discord, etc.)]**	**LUGAR (2/3)**<br><br>**¿Dónde sucedió?**<br><br>**[OPCIONES: jugando un juego de jugadorxs múltiples con amigxs cercanxs y/o familia, jugando un juego de jugadorxs múltiples con conocidxs y/o extrańxs, socializando en una comunidad de jugadorxs (ej., Twitch, Discord, etc.]**
	**Someone said or implied...**	**Alguien dijo (o implicó)...**
	being LGBTQ+ is a "just a phase."	ser LGBTQ+ es "nada más una fase."
	it is a choice to be LGBTQ+.	es una elección ser LGBTQ+.
	an anti-LGBTQ+ slur (e.g., "fag," "queer").	un insulto anti-LGBTQ+ (ej., "puto" "maricón).
	a negative LGBTQ+ joke.	un chiste contra lo LGBTQ+.
	that you/others were overly sensitive for thinking that you/they were being treated unfairly because of your or their LGBTQ+ identity.	que tú/otrxs eran súmamente sensiblxs por pensar que tú/ellxs estaban siendo tratadxs de una manera injusta por su/tu identidad LGBTQ+.
	the wrong personal pronouns or name to reference you/others.	los pronombres equivocados, o el nombre equivocado para hablar de tí o de otrxs.
	that an anti-LGBTQ+ comment didn't mean anything, or it was just a joke.	que un comentario anti-LGBTQ+ no significaba nada o era solo un chiste.
	that you/others were overreacting in response to an anti-LGBTQ+ joke (e.g., called them a snowflake or over-sensitive).	que tú/otrxs estaban sobre-reaccionando en respuesta a un chiste anti-LGBTQ+ (que les llamaran sobre-sensible o ridículx).
	"that's how the real world is" in a conversation about anti-LGBTQ+ prejudice and discrimination.	"así es el mundo" en una conversación sobre prejuicios y discriminación contra lo LGBTQ+
	that you/others should stop complaining about anti-LGBTQ+ prejudice and discrimination.	que tú/otrxs deberían dejar de quejarse sobre prejuicios y discriminación contra lo LGBTQ+
	that it is wrong to be LGBTQ+ or that you/others will go to hell because of your/others' LGBTQ+ identity.	que está mal ser LGBTQ+ o que tú/otrxs se irán al infierno por la identidad LGBT que tienen

	that LGBTQ+ is a result of something that went “wrong” in your/others’ past (e.g., “mother was too overbearing,” “father wasn’t around”).	que ser LGBTQ+ es resultado de que algo “salió mal” en tu pasado o el de otrxs (ej., “tu mamá fue muy dominante”, o “tu padre no estuvo presente").
	that they were praying for you/others because they knew or assumed that you/others were LGBTQ+.	que estaban rezando por tí/otrxs porque saben o asumen que eres/son LGBTQ+.
	that you/others would hit on them because of your LGBTQ+ identity.	que tú/otrxs se insinuarían sexualmente a ellxs debido a su identidad LGBTQ+.
**42**	**FREQUENCY (3/3)**<br><br>**How often have you experienced/witnessed the following events?**<br><br>**[OPTIONS: never, rarely (e.g., less than once a month), sometimes (e.g., 1-3 times a month), often (e.g., 1-3 times a week), daily.]**	**FRECUENCIA (3/3)**<br><br>**¿Qué tan seguido has experimentado/sido testigo de esto?**<br><br>**[OPCIONES: nunca, casi nunca (ej., menos de una vez al mes), a veces (ej., 1-3 veces al mes), seguido (ej., 1-3 times a week), diario.]**
	**Someone said or implied...**	**Alguien dijo (o implicó)...**
	being LGBTQ+ is a “just a phase.”	ser LGBTQ+ es “nada más una fase.”
	it is a choice to be LGBTQ+.	es una elección ser LGBTQ+.
	an anti-LGBTQ+ slur (e.g., “fag,” “queer”).	un insulto anti-LGBTQ+ (ej., “puto” “maricón).
	a negative LGBTQ+ joke.	un chiste contra lo LGBTQ+.
	that you/others were overly sensitive for thinking that you/they were being treated unfairly because of your or their LGBTQ+ identity.	que tú/otrxs eran súmamente sensiblxs por pensar que tú/ellxs estaban siendo tratadxs de una manera injusta por su/tu identidad LGBTQ+.
	the wrong personal pronouns or name to reference you/others.	los pronombres equivocados, o el nombre equivocado para hablar de tí o de otrxs.
	that an anti-LGBTQ+ comment didn’t mean anything, or it was just a joke.	que un comentario anti-LGBTQ+ no significaba nada o era solo un chiste.
	that you/others were overreacting in response to an anti-LGBTQ+ joke (e.g., called them a snowflake or over-sensitive).	que tú/otrxs estaban sobre-reaccionando en respuesta a un chiste anti-LGBTQ+ (que les llamaran sobre-sensible o ridículx).
	“that’s how the real world is” in a conversation about anti-LGBTQ+ prejudice and discrimination.	"así es el mundo” en una conversación sobre prejuicios y discriminación contra lo LGBTQ+

	that you/others should stop complaining about anti-LGBTQ+ prejudice and discrimination.	que tú/otrxs deberían dejar de quejarse sobre prejuicios y discriminación contra lo LGBTQ+
	that it is wrong to be LGBTQ+ or that you/others will go to hell because of your/others' LGBTQ+ identity.	que está mal ser LGBTQ+ o que tú/otrxs se irán al infierno por la identidad LGBT que tienen
	that LGBTQ+ is a result of something that went "wrong" in your/others' past (e.g., "mother was too overbearing," "father wasn't around").	que ser LGBTQ+ es resultado de que algo "salió mal" en tu pasado o el de otrxs (ej., "tu mamá fue muy dominante", o "tu padre no estuvo presente").
	that they were praying for you/others because they knew or assumed that you/others were LGBTQ+.	que estaban rezando por tí/otrxs porque saben o asumen que eres/son LGBTQ+.
	that you/others would hit on them because of your/their LGBTQ+ identity.	que tú/otrxs se insinuarían sexualmente a ellxs debido a tu/su identidad LGBTQ+.
**46**	**EXPERIENCE (1/3)**<br><br>**Instructions**: For each of the following, please indicate whether the experience described has happened to you, happened to others, is a regular and accepted part of gaming culture, or has never happened while playing multiplayer games (including voice or text chat) or when using gaming communities and spaces (for example, Twitch, Discord, Reddit, etc.).<br><br>These questions will span multiple pages.<br><br>**Content warning:** The following items ask you to recall homophobic and/or transphobic instances that you may have experienced or observed while playing video games. There is mention of slurs that have been used to harm LGBTQ+ people. Please take a minute to take a break or take care of your wellbeing.<br><br>**[OPTIONS: this happened to me, I saw this happen to others, this feels like an accepted part of gaming culture, I haven't experienced or witnessed this]**	**EXPERIENCIA (1/3)**<br><br>**Instrucciones:** Para cada una de las siguientes, por favor indica si la experiencia descrita te sucedió a tí, le sucedió a otrxs, es una parte regular y aceptada de la cultura de los videojuegos o nunca ha pasado al jugar juegos con jugadorxs múltiples (incluyendo voz o chat por texto) o cuando se usan comunidades o espacios de juego (por ejemplo, Twitch, Discord, Reddit, etc.)<br><br>Estas preguntas abarcarán varias páginas.<br><br>**Advertencia de contenido:** Los siguientes elementos piden que recuerdes instancias homofóbicas y/o transfóbicas que pudiste haber experimentado u observado al jugar videojuegos. Hay mención de insultos que han sido usados para hacerle daño a las personas LGBTQ+. Por favor tómate un minuto para respirar o cuidar tu bienestar.<br><br>**[OPCIONES: esto me sucedió a mí, vi que esto le pasó a otrxs, esto se siente como una parte aceptada de la comunidad de los videojuegos, no he experimentado ni he sido testigo de esto.]**

	Someone stopped talking to you/others after finding out about your/their LGBTQ+ identity.	Alguien dejó de hablar contigo al enterarse de tu identidad LGBTQ+ o la de otrxs.
	Someone seemed willing to tolerate LGBTQ+ identity but didn't seem willing to talk about it.	Alguien parece dispuesto a tolerar las identidades LGBTQ+ pero no parece dispuesto a hablar de ello.
	Someone changed the subject/topic when LGBTQ+ identity came up.	Alguien cambió el tema cuando salieron a la conversación las identidades LGBTQ+.
**47**	**SETTING (2/3)**<br><br>**Please indicate where the event(s) happened**<br><br>**[OPTIONS: playing a multiplayer game with close friends or family, playing a multiplayer game with acquaintances and/or strangers, in a gaming community (i.e., Twitch, Discord, etc.)]**	**LUGAR (2/3)**<br><br>**¿Dónde sucedió?**<br><br>**[OPCIONES: jugando un juego de jugadorxs múltiples con amigxs cercanxs y/o familia, jugando un jego de jugadorxs múltiples con conocidxs y/o extrańxs, socializando en una comunidad de jugadorxs (ej., Twitch, Discord, etc.]**
	Someone stopped talking to you/others after finding out about your/their LGBTQ+ identity.	Alguien dejó de hablar contigo al enterarse de tu identidad LGBTQ+ o la de otrxs.
	Someone seemed willing to tolerate LGBTQ+ identity but didn't seem willing to talk about it.	Alguien parece dispuesto a tolerar las identidades LGBTQ+ pero no parece dispuesto a hablar de ello.
	Someone changed the subject/topic when LGBTQ+ identity came up.	Alguien cambió el tema cuando salieron a la conversación las identidades LGBTQ+.
**48**	**FREQUENCY (3/3)**<br><br>**How often have you experienced/witnessed the following events?**<br><br>**[OPTIONS: never, rarely (e.g., less than once a month), sometimes (e.g., 1-3 times a month), often (e.g., 1-3 times a week), daily.]**	**FRECUENCIA (3/3)**<br><br>**¿Qué tan seguido has experimentado/sido testigo de esto?**<br><br>**[OPCIONES: nunca, casi nunca (ej., menos de una vez al mes), a veces (ej., 1-3 veces al mes), seguido (ej., 1-3 times a week), diario.]**
	Someone stopped talking to you/others after finding out about your/their LGBTQ+ identity.	Alguien dejó de hablar contigo al enterarse de tu identidad LGBTQ+ o la de otrxs.
	Someone seemed willing to tolerate LGBTQ+ identity but didn't seem willing to talk about it.	Alguien parece dispuesto a tolerar las identidades LGBTQ+ pero no parece dispuesto a hablar de ello.

	Someone changed the subject/topic when LGBTQ+ identity came up.	Alguien cambió el tema cuando salieron a la conversación las identidades LGBTQ+.
**49**	What video games or communities do negative experiences most often occur within?	¿En qué videojuegos o comunidades ocurren con mayor frecuencia experiencias negativas?
**50**	**Optional:** Please share more about any **negative experiences** you have had related to being LGBTQ+ when playing video games online (including audio or text in game chat).	**Opcional:** Por favor comparte lo más que quieras sobre experiencias negativas que has tenido relacionado con ser LGBTQ+ al jugar videojuegos en línea (incluyendo audio o texto en el chat del juego).

	**SECTION 4.2: IMPACT OF NEGATIVE EXPERIENCES IN MULTIPLAYER GAMES, GAMING COMMUNITIES/SPACES & STREAMS**	**SECCIÓN 4.2: IMPACT OF EXPERIENCIAS NEGATIVAS JUGANDO JUEGOS DE JUGADORXS MÚLTIPLES, USANDO COMUNIDADES Y ESPACIOS DE JUEGO, Y O JUEGOS CON STREAMING**
**50**	**Instructions:** Thinking about all of the **negative** experiences with **multiplayer games, gaming communities, and streams**, how much were you impacted in the following ways?<br><br>**RATING SCALE:** (0 – 4). [The default is at 0].<br>I was not affected this way at all; I was affected this way a little bit; I was somewhat affected this way; I was affected this way quite a bit; I was very much affected this way	**Instrucciones:** Pensando en todas las experiencias **negativas** con los **juegos multijugador, las comunidades de juegos y streams en linéa,** ¿cuánto te impactó de las siguientes maneras?<br><br>**ESCALA DE RANGO:** (0 – 4). [El default está en 0].<br>No me afectó en lo absoluto; me afectó de esta manera un poco; me afectó algo de esta manera; me afectó de esta manera bastante; me afectó mucho de esta manera.
	I felt angry.	Me sentí enojadx.
	I felt frustrated.	Me sentí frustradx.
	I felt upset.	Me sentí molestx.
	I was in shock.	Estaba en shock.
	I felt like I had no control.	Sentí que perdía control.
	I felt embarrassed.	Me sentí avergonzadx.
	I felt anxious.	Me sentí ansiosx.
	I felt worthless.	Me sentí inútil.
	I felt lost.	Me sentí perdidx.
	I stopped playing the game.	Dejé de jugar el juego.
	I blocked someone.	Bloqueé a alguien.
	I deleted/uninstalled the game.	Eliminé / desinstalé el juego.

	I played the game less.	Jugué menos al juego.
	I went to see a psychologist/counselor.	Fui a ver a un psicólogx / consejerx.
	I got (or tried to get) support from a friend or other trusted adult.	Recibí (o traté de obtener) el apoyo de un amigx u otro adultx de confianza.
	I felt worse about being an LGBTQ+ person.	Me sentí peor por ser una persona LGBTQ+.
	I felt better about being an LGBTQ+ person.	Me sentí mejor por ser una persona LGBTQ+.
	I was ***less*** likely to talk about being LGBTQ+ with people **ONline**.	Era ***menos*** probable que hablara sobre ser LGBTQ + con personas **en línea**.
	I was ***less*** likely to talk about being LGBTQ+ with people **OFFline**.	Era ***menos*** probable que hablara sobre ser LGBTQ + **en persona**.
	I was ***more*** likely to talk about being LGBTQ+ with people **ONline**.	Era ***más*** probable que hablara sobre ser LGBTQ + con personas **En línea**.
	I was ***more*** likely to talk about being LGBTQ+ with people **OFFline**.	Era ***más*** probable que hablara sobre ser LGBTQ + **en persona**.
	I feel like I have become a better person.	Siento que me he convertido en una mejor persona.
	I learned more about myself.	Aprendí más sobre mí mismo.
	It made me realise what was really important to me.	Me hizo darme cuenta de lo que era realmente importante para mí.
	I felt like a stronger person after the experience.	Me sentí como una persona más fuerte después de la experiencia.
	My experience made me more sensitive to others who have similar experiences.	Mi experiencia me hizo más sensible a otros que tienen experiencias similares.
**51**	**Optional:** Please share more about **the impacts** of any **negative** experiences you have had in multiplayer games, gaming communities/spaces, and during streaming. [WRITE IN RESPONSE]	**Opcional:** Por favor comparte más acerca de **los impactos** de cualquier experiencia negativa que has tenido relacionado con ser LGBTQ+ en **juegos multijugador, las comunidades de juegos y streams en linéa**. [ESCRIBE TU RESPUESTA]

	**SECTION 5.1: POSITIVE EXPERIENCES IN MULTIPLAYER GAMES, GAMING COMMUNITIES/SPACES & STREAMS**	**SECCIÓN 5.1: EXPERIENCIAS POSITIVAS JUGANDO JUEGOS DE JUGADORXS MÚLTIPLES, USANDO COMUNIDADES Y ESPACIOS DE JUEGO, Y O JUEGOS CON STREAMING**
**52**	**EXPERIENCE (1/3)**<br><br>**Instructions:** For each of the following, please indicate whether the experience described has happened to you, happened to others, is a regular and accepted part of gaming culture, or has never happened while playing multiplayer games (including voice or text chat) or when using gaming communities and spaces (for example, Twitch, Discord, Reddit, etc.).<br><br>**[OPTIONS: this happened to me, I saw this happen to others, this feels like an accepted part of gaming culture, I haven't experienced or witnessed this]**	**EXPERIENCIA (1/3)**<br><br>**Instrucciones:** Para cada uno de los siguientes, indique si la experiencia descrita le ha sucedido a usted, le ha sucedido a otros, es una parte regular y aceptada de la cultura de juego, o nunca ha sucedido mientras jugaba juegos multijugador (incluyendo voz o chat por texto) o cuando se usan comunidades y espacios de juego (por ejemplo, Twitch, Discord, Reddit, etc.).<br><br>**[OPCIONES: esto me sucedió a mí, vi que esto le pasó a otrxs, esto se siente como una parte aceptada de la comunidad de los videojuegos, no he experimentado ni he sido testigo de esto.]**
	Someone was supportive after learning about my/others' LGBTQ+ identity.	Alguien brindó apoyo después de enterarse sobre mi identidad LGBTQ+ o la de otrxs.
	Someone asked what pronouns they should use for me/another person.	Alguien preguntó qué pronombres debe usar para mí o otrxs.
	Someone shared about or sent an invite to join an LGBTQ+-affirming gaming community (e.g., Discord server).	Alguien te compartió o te/a otrxs envió una invitación para unirte a una comunidad de juego LGBTQ+ (ej., un servidor de Discord).
	Someone corrected themself when they used the incorrect pronoun or name to reference me/others.	Alguien se corrigió al usar pronombres incorrectos para referirse a mí/otrxs.

	Someone spoke positively about LGBTQ+ people.	Alguien habló de forma positiva sobre las personas LGBTQ+ contigo o con otrxs.
	Someone stepped in to offer support after others made anti-LGBTQ+ comments or jokes.	Alguien se involucró para ofrecer apoyo después de que otras personas hicieran comentarios o chiste anti-LGBTQ+.
	Someone was welcoming and inclusive towards me/others.	Alguien me hizo sentir bienvenidx e incluídx.
	Someone was non-judgmental about me/others being LGBTQ+.	Alguien no me juzgó por ser LGBTQ+.
	Someone took a sincere interest in LGBTQ+ topics when talking to me/others.	Alguien tuvo un interés sincero por temas LGBTQ+.
**53**	**SETTING (2/3)**<br><br>**Please indicate where the event(s) happened**<br><br>**[OPTIONS: playing a multiplayer game with close friends or family, playing a multiplayer game with acquaintances and/or strangers, in a gaming community (i.e., Twitch, Discord, etc.)]**	**LUGAR (2/3)**<br><br>**¿Dónde sucedió?**<br><br>**[OPCIONES: jugando un juego de jugadorxs múltiples con amigxs cercanxs y/o familia, jugando un jego de jugadorxs múltiples con conocidxs y/o extrańxs, socializando en una comunidad de jugadorxs (ej., Twitch, Discord, etc.]**
	Someone was supportive after learning about my/others' LGBTQ+ identity.	Alguien brindó apoyo después de enterarse sobre mi identidad LGBTQ+ o la de otrxs.
	Someone asked what pronouns they should use for me/another person.	Alguien preguntó qué pronombres debe usar para mí o otrxs.
	Someone shared about or sent an invite to join an LGBTQ+-affirming gaming community (e.g., Discord server).	Alguien te compartió o te/a otrxs envió una invitación para unirte a una comunidad de juego LGBTQ+ (ej., un servidor de Discord).
	Someone corrected themself when they used the incorrect pronoun or name to reference me/others.	Alguien se corrigió al usar pronombres incorrectos para referirse a mí/otrxs.
	Someone spoke positively about LGBTQ+ people.	Alguien habló de forma positiva sobre las personas LGBTQ+ contigo o con otrxs.
	Someone stepped in to offer support after others made anti-LGBTQ+ comments or jokes.	Alguien se involucró para ofrecer apoyo después de que otras personas hicieran comentarios o chiste anti-LGBTQ+.
	Someone was welcoming and inclusive towards me/others.	Alguien me hizo sentir bienvenidx e incluídx.
	Someone was non-judgmental about me/others being LGBTQ+.	Alguien no me juzgó por ser LGBTQ+.

	Someone took a sincere interest in LGBTQ+ topics when talking to me/others.	Alguien tuvo un interés sincero por temas LGBTQ+.
**54**	**FREQUENCY (3/3)**<br><br>**How often have you experienced/witnessed the following events?**<br><br>**[OPTIONS: never, rarely (e.g., less than once a month), sometimes (e.g., 1-3 times a month), often (e.g., 1-3 times a week), daily.]**	**FRECUENCIA (3/3)**<br><br>**¿Qué tan seguido has experimentado/sido testigo de esto?**<br><br>**[OPCIONES: nunca, casi nunca (ej., menos de una vez al mes), a veces (ej., 1-3 veces al mes), seguido (ej., 1-3 times a week), diario.]**
	Someone was supportive after learning about my/others' LGBTQ+ identity.	Alguien brindó apoyo después de enterarse sobre mi identidad LGBTQ+ o la de otrxs.
	Someone asked what pronouns they should use for me/another person.	Alguien preguntó qué pronombres debe usar para mí o otrxs.
	Someone shared about or sent an invite to join an LGBTQ+-affirming gaming community (e.g., Discord server).	Alguien te compartió o te/a otrxs envió una invitación para unirte a una comunidad de juego LGBTQ+ (ej., un servidor de Discord).
	Someone corrected themself when they used the incorrect pronoun or name to reference me/others.	Alguien se corrigió al usar pronombres incorrectos para referirse a mí/otrxs.
	Someone spoke positively about LGBTQ+ people.	Alguien habló de forma positiva sobre las personas LGBTQ+ contigo o con otrxs.
	Someone stepped in to offer support after others made anti-LGBTQ+ comments or jokes.	Alguien se involucró para ofrecer apoyo después de que otras personas hicieran comentarios o chiste anti-LGBTQ+.
	Someone was welcoming and inclusive towards me/others.	Alguien me hizo sentir bienvenidx e incluídx.
	Someone was non-judgmental about me/others being LGBTQ+.	Alguien no me juzgó por ser LGBTQ+.
	Someone took a sincere interest in LGBTQ+ topics when talking to me/others.	Alguien tuvo un interés sincero por temas LGBTQ+.
**55**	What video games or communities do	
**56**	**Optional:** Please share more about any positive experience you have had while playing multiplayer video games (includes audio or text in game chat), by using gaming communities (for example, Twitch, Discord, Reddit), or streaming gameplay.	**Opcional:** Por favor comparte más acerca sobre experiencias positivas que has tenido al jugar juegos de jugadorxs múltiples (incluye audio o texto por el chat del juego), al usar comunidades de juego (por ejemplo, Twitch, Discord, Reddit) o juego por streaming.

	SECTION 5.2: IMPACT OF POSITIVE EXPERIENCES IN MULTIPLAYER GAMES, GAMING COMMUNITIES/SPACES & STREAMS	SECCIÓN 5.2: IMPACTO DE LAS EXPERIENCIAS POSITIVAS JUGANDO JUEGOS DE JUGADORXS MÚLTIPLES, USANDO COMUNIDADES Y ESPACIOS DE JUEGO, Y O JUEGOS CON STREAMING
**57**	**Instructions:** Thinking about all of the positive experiences in **multiplayer games, gaming communities/spaces, and streams**, how much were you impacted in the following ways?<br><br>**RATING SCALE:** (0 – 4). [The default is at 0].<br>I was not affected this way at all; I was affected this way a little bit; I was somewhat affected this way; I was affected this way quite a bit; I was very much affected this way	**Instrucciones:** Pensando en todas las experiencias positivas en juegos **multijugador, comunidades / espacios de juego y transmisiones,** ¿cuánto se vio afectado de las siguientes maneras?<br><br>**ESCALA DE RANGO:** (0 – 4). [El default está en 0].<br>No me afectó en lo absoluto; me afectó de esta manera un poco; me afectó algo de esta manera; me afectó de esta manera bastante; me afectó mucho de esta manera.
	I felt happy.	Me sentí feliz.
	I felt supported.	Me sentí apoyadx.
	I felt comfortable.	Me sentí cómodx.
	I felt like part of a group.	Me sentí como parte de un grupo.
	I felt less stress.	Sentí menos estrés.
	I felt less anxiety.	Sentí menos ansiedad.
	I played the game more.	Jugué más el juego.
	I added or friended someone.	Agregué a alguien como amigx.
	I played the game less.	Jugué menos el juego.
	I felt worse about being an LGBTQ+ person.	Me sentí peor por ser una persona LGBTQ+.
	I felt better about being an LGBTQ+ person.	Me sentí mejor por ser una persona LGBTQ+.
	I was ***less*** likely to talk about being LGBTQ+ with people **ONline**.	Estoy ***menos*** cómodx con hablar acerca de ser LGBTQ+ con personas **en línea**.
	I was ***less*** likely to talk about being LGBTQ+ with people **OFFline**.	Estoy ***menos*** cómodx con hablar acerca de ser LGBTQ+ **en persona**.

	I was ***more*** likely to talk about being LGBTQ+ with people **ONline**.	Estoy ***más*** cómodx con hablar acerca de ser LGBTQ+ con personas **en línea**.
	I was ***more*** likely to talk about being LGBTQ+ with people **OFFline**.	Estoy ***más*** cómodx con hablar acerca de ser LGBTQ+ **en persona**.
	I feel like I have become a better person.	Siento que me convertido en mejor persona.
	I learned more about myself.	Aprendí más acerca de mí mismx.
	It made me realize what was really important to me.	Me hizo darme cuenta de lo que realmente es importante para mí.
	I learned to stand up for myself.	Aprendí a defenderme.
	I learned not to care as much about what people think about me.	Aprendí a que me importara menos lo que la gente piensa de mí.
	I felt like a stronger person after the experience.	Me sentí como una persona más fuerte después de la experiencia.
	My experience made me more sensitive to others who have similar experiences.	Mi experiencia me hizo más sensible a otrxs que han tenido experiencias similares.
**58**	**Optional**: Please share more about **the impacts** of any **positive** experiences you've had related to being LGBTQ+ during **multiplayer games, while using gaming communities and spaces, and/or while streaming**. [WRITE IN RESPONSE]	**Opcional:** Comparte más sobre **los impactos** de cualquier **experiencias positivas** que hayas tenido relacionada con ser LGBTQ + **jugando juegos de jugadorxs múltiples, usando comunidades y espacios de juego, y/o streaming en linéa.** [ESCRIBE TU RESPUESTA]

SECTION 6: SURVEY END	SECCIÓN 6: FIN DE LA ENCUESTA
By participating in the LEVEL UP! Project, you are helping us to make gaming a more open place for LGBTQ+ youth!<br><br>For participating in this research, we would like to thank you by offering you an opportunity to participate in a raffle to win one of 10 $50 CAD (40 USD; 30 GBP; 55 AUD; 819 MXN) Amazon gift cards. More information will be available after you submit the survey.<br><br>Are you interested in participating in the raffle?<br>a) Yes **[LINK TO A SEPARATE SURVEY ON ANOTHER PAGE]**<br>b) No **[END SURVEY]**	Al participar en el proyecto ¡SUBÉ DE NIVEL!, ¡estás ayudándonos a hacer que los espacios de videojuegos sean más abiertos para las juventudes LGBTQ+!<br><br>Por participar en esta investigación, nos gustaría darte las gracias al ofrecerte una oportunidad para participar en una rifa para una tarjeta de regalo con valor de $25 dólares canadienses para una tienda de videojuegos de tu elección (PlayStation, Xbox, Nintendo Switch, Steam, o iTunes). Más información estará disponible después de que entregues la encuesta.<br><br>¿Estás interesado en participar en el sorteo?<br>**a)** Sí **[LIGA A UNA ENCUESTA SEPARADA EN OTRA PÁGINA]**<br>**b)** No **[TERMINAR LA ENCUESTA]**
**SEPARATE SURVEY**<br><br>What country do you live in?<br>a) Australia<br>b) Canada<br>c) Mexico<br>d) United Kingdom<br>e) United States<br><br>Escribe tu dirección de correo electrónico. Nos pondremos en contacto contigo si ganas el sorteo. [WRITE IN RESPONSE]	**ENCUESTA SEPARADA**<br><br>¿En qué país vives?<br>a) Australia<br>b) Canadá<br>c) México<br>d) Reino Unido<br>e) Estados Unidos<br><br>Escribe tu dirección de correo electrónico. Nos pondremos en contacto contigo si ganas el sorteo. [ESCRIBE TU RESPUESTA]
